# Dose-Dependent Nuclear Delivery and Transcriptional Repression with a Cell-Penetrant MeCP2

**DOI:** 10.1021/acscentsci.2c01226

**Published:** 2023-02-03

**Authors:** Xizi Zhang, Claudia Cattoglio, Madeline Zoltek, Carlo Vetralla, Deepto Mozumdar, Alanna Schepartz

**Affiliations:** † Department of Chemistry, University of California, Berkeley, California 94720, United States; ‡ Department of Molecular and Cellular Biology, University of California, Berkeley, California 94720, United States; § Howard Hughes Medical Institute, University of California, Berkeley, California 94720, United States; ∥ Department of Chemistry, Yale University, New Haven, Connecticut 06520, United States; ⊥ California Institute for Quantitative Biosciences (QB3), University of California, Berkeley, California 94720, United States; # Chan Zuckerberg Biohub, San Francisco, California 94158, United States

## Abstract

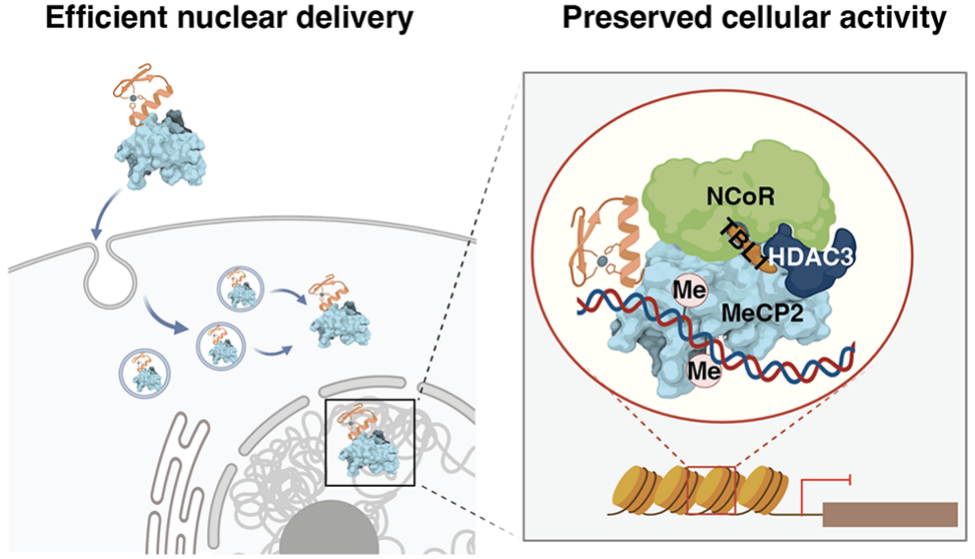

The vast majority of biologic-based therapeutics operate within serum, on the cell surface, or within endocytic vesicles, in large part because proteins and nucleic acids fail to efficiently cross cell or endosomal membranes. The impact of biologic-based therapeutics would expand exponentially if proteins and nucleic acids could reliably evade endosomal degradation, escape endosomal vesicles, and remain functional. Using the cell-permeant mini-protein ZF5.3, here we report the efficient nuclear delivery of functional Methyl-CpG-binding-protein 2 (MeCP2), a transcriptional regulator whose mutation causes Rett syndrome (RTT). We report that ZF-*t*MeCP2, a conjugate of ZF5.3 and MeCP2(Δaa13–71, 313–484), binds DNA in a methylation-dependent manner *in vitro*, and reaches the nucleus of model cell lines intact to achieve an average concentration of 700 nM. When delivered to live cells, ZF-*t*MeCP2 engages the NCoR/SMRT corepressor complex, selectively represses transcription from methylated promoters, and colocalizes with heterochromatin in mouse primary cortical neurons. We also report that efficient nuclear delivery of ZF-*t*MeCP2 relies on an endosomal escape portal provided by HOPS-dependent endosomal fusion. The Tat conjugate of MeCP2 (Tat-*t*MeCP2), evaluated for comparison, is degraded within the nucleus, is not selective for methylated promoters, and trafficks in a HOPS-independent manner. These results support the feasibility of a HOPS-dependent portal for delivering functional macromolecules to the cell interior using the cell-penetrant mini-protein ZF5.3. Such a strategy could broaden the impact of multiple families of biologic-based therapeutics.

## Abstract 2

The cell-permeant mini-protein ZF5.3 efficiently delivers the therapeutically relevant transcriptional regulator MeCP2 to the nucleus of model cells and primary neurons while preserving protein function.

## Introduction

The efficient delivery of proteins and nucleic acids to the cytosol and internal organelles remains a significant and unmet challenge in an era exploding with novel therapeutic strategies. The vast majority of FDA-approved biologics are delivered via injection, and operate within serum, on cell surfaces, or within endosomal vesicles. Those that do operate in the cytosol^1−5^ notably achieve activity with endosomal escape efficiencies of 10% or lower.^6,7^ The impact of biologic-based therapeutic strategies would expand exponentially if proteins and nucleic acids could reliably evade endosomal degradation, escape endosomal vesicles, and remain functional. Yet progress toward this goal has been exceptionally slow. Most delivery strategies^8^ are inefficient,^9^ rely on degradation-prone molecular scaffolds,^10^ operate via undefined or multifarious mechanisms,^11^ and evaluate activity using amplified or indirect assays.^9,12^

We showed previously that the mini-protein ZF5.3^9,13−16^ is taken up by the endosomal pathway and released efficiently into the cytosol and nucleus of live cells,^14^ alone and when fused to certain protein cargos. Proteins successfully delivered using ZF5.3 include the self-labeling protein SNAP-tag,^9^ the metabolic enzyme argininosuccinate synthetase,^16^ the proximity labeling tool APEX2,^9^ and a nanobody-based degrader.^17^ These proteins differ in molecular weight, stoichiometry, isoelectric point, and the presence of bound cofactors. In all cases evaluated,^9,16^ the protein that reached the cytosol was fully intact as judged by Western blot analysis of isolated cytosolic fractions, and the delivery efficiencies were 2–10-fold^9^ higher than seen with canonical or cyclic peptides.^18^ Mechanistic studies confirm that ZF5.3 relies on the endocytic pathway to reach the cell interior,^13^ and that endosomal escape into the cytosol demands a functional homotypic fusion and protein sorting (HOPS) complex.^15^

Methyl-CpG-binding-protein 2 (MeCP2) is an abundant nuclear protein expressed in all cell types, especially neurons.^19^ Mutations in the X-chromosome-linked *MECP2* gene cause Rett syndrome (RTT), a severe and incurable neurological disorder that disproportionately affects young girls.^20^ Many potential RTT treatments are under development,^21^ but no disease modifying treatment yet exists. Two features of RTT etiology render therapeutic development especially challenging. The first is that more than 850 different mutations in the *MECP2* gene^22^ account for >95% of classical RTT cases;^23^ this feature complicates approaches based on gene-editing.^24−26^ The second is toxicity caused by overexpression of MeCP2; this feature complicates approaches that rely on gene delivery.^27−31^ Since 2007, several studies have demonstrated that restoring 70–80% of the native levels of MeCP2 protein could alleviate neurological symptoms of RTT, restore neuronal signaling, and greatly improve the survival rate in male and female MeCP2-deficient mice.^32−34^ These rescue experiments support the hypothesis that dose-dependent, nuclear delivery of functional MeCP2 protein could provide a novel treatment modality. Although the concentration of MeCP2 varies between cell types, its primary function is to regulate gene transcription. MeCP2 can repress gene expression by engaging the NCoR/SMRT corepressor complex in a methylated DNA-dependent manner.^35^ By interacting with CREB1, it can also function as a transcription activator.^36^ In order to be effective, MeCP2 protein must reach the nucleus intact, transcriptionally active, and in the high nanomolar to low micromolar concentration range.^19,37^

Previous efforts to deliver MeCP2 protein were largely ineffective, in large part because of low delivery efficiencies and significant levels of cargo degradation. A cell-permeable nanobody functionalized with a cyclic arginine-rich peptide (cR_10_) was reported to deliver a MeCP2-GFP fusion protein to the nucleus with low efficiency (2–10%) after cells were treated with high concentrations (20 μM) of the fusion.^38^ A conjugate between MeCP2 and residues 47–57 of the HIV-1 transactivator of transcription protein Tat (Tat-MeCP2) reached the interior of human fibroblasts^10,39,40^ and primary mouse neurons^10^ as determined by confocal microscopy and Western blots, reversed histone hyperacetylation, and interacted with the partner protein HDAC1.^10,41^ In this case, however, long incubation times (>12 h) were needed, the established nuclear concentration was low (∼4 nM),^39^ and significant degradation was observed.^10^

Here we use chemical biology, cell biology, biophysics, and biochemistry tools to qualitatively and quantitatively assess the nuclear delivery and function of MeCP2 conjugates of ZF5.3 and Tat. Although both conjugates bind DNA in a methylation-dependent manner *in vitro* and appear to reach the nucleus as judged by fluorescence-based methods, biochemical fractionation studies reveal that only the conjugate with ZF5.3 remains fully intact within the nucleus. When delivered to live cells, the conjugate between ZF5.3 and MeCP2 effectively engages the NCoR/SMRT corepressor complex, selectively represses transcription from methylated promoters, and colocalizes with heterochromatin in mouse primary cortical neurons. Efficient nuclear delivery relies on HOPS-dependent endosomal fusion. By contrast, the Tat conjugate of MeCP2 is degraded within the nucleus, is not selective for methylated promoters, and trafficks in a HOPS-independent manner. The results described here support the feasibility of a HOPS-dependent portal for delivering functional macromolecules to the cell interior using the cell-penetrant mini-protein ZF5.3. Such a strategy could broaden the impact of multiple families of biologic-based therapeutics.

## Results

### Design, Purification, and Characterization of MeCP2 Variants

**Figure 1:**
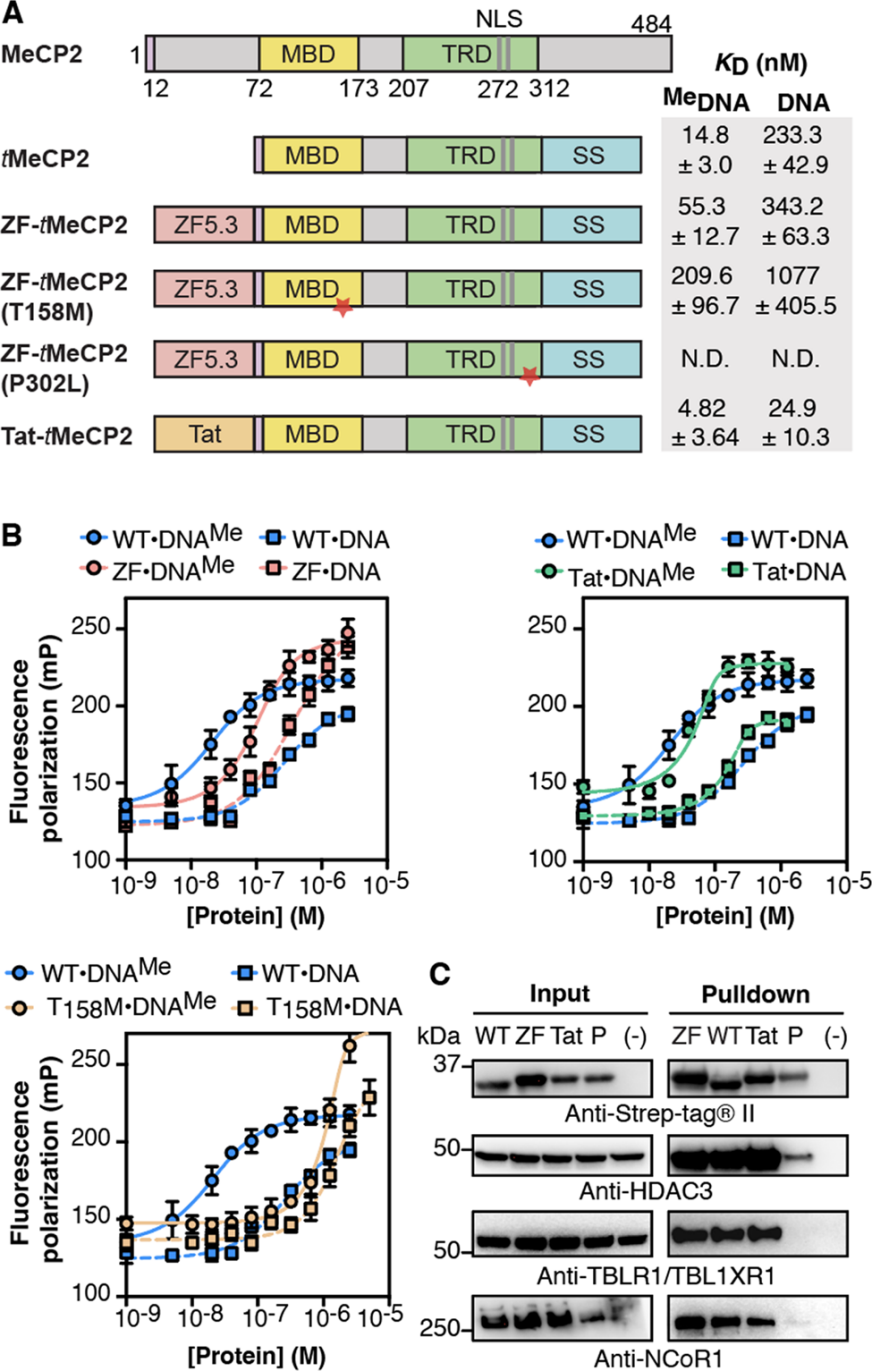
ZF-*t*MeCP2 is functional *in vitro*. (A) *t*MeCP2 proteins used in this work lack N-terminal residues 13–71 and C-terminal residues 313–484. All proteins were expressed in *E. coli* and purified as described in Supplementary Methods 1. We note that as the *t*MeCP2 proteins used in this work were expressed in bacteria, they lack post-translational arginine modifications that may affect heterochromatin accumulation, clustering and binding kinetics.^50^ MBD: methyl-CpG-binding domain; TRD: transcriptional-repressor domain; NLS: nuclear localization sequence; SS: sortase motif + Strep-tag. The red star indicates the location of the point mutation. (B) Plots showing changes in fluorescence polarization used to calculate the apparent equilibrium dissociation constant (*K*_D_) of the complex between each *t*MeCP2 variant and methylated (DNA^Me^) or nonmethylated (DNA) oligonucleotides (Table S5). The data were fitted to an equilibrium binding equation based on the Langmuir model^51^ to calculate the *K*_D_ values in (A). Data are represented as mean ± SD. N.D.: not determined. (C) Western blots were used to analyze an *in vitro* anti-Strep-tag pull-down assay to probe the interaction of the indicated *t*MeCP2 variant (WT: *t*MeCP2, ZF: ZF-*t*MeCP2, Tat: Tat-*t*MeCP2, P: ZF-*t*MeCP2(P302L)) with subunits of the NCoR/SMRT repressor complex. HDAC3: Histone Deacetylase 3; TBLXR1: Transducin Beta-Like 1X-Related Protein 1; NCoR1: Nuclear Receptor Corepressor 1. The gel results shown are representative of three biological replicates.

Full-length murine MeCP2 (MeCP2-e2, protein sequence identifier: Q9Z2D6–1) contains 484 amino acids (52 kDa) (Figure 1A).^28^ MeCP2(ΔNC) (referred to henceforth as *t*MeCP2) is shorter (27 kDa, 253 aa) but mirrors MeCP2 in its interactions with methylated DNA and the NCoR/SMRT complex, and *Mecp2*-null male mice display a near-normal phenotype upon expression of MeCP2(ΔNC).^28^ We generated fusion proteins containing a single copy of ZF5.3^13^ or Tat_47–57_^42^ followed by the complete sequence of *t*MeCP2 (Figure 1A). Each fusion protein also contained a sortase recognition motif (6 aa) to enable site-specific fluorophore conjugation and a Strep-tag II sequence (8 aa) to enable affinity purification. We also prepared two *t*MeCP2 variants with substitutions that alter function. The first is T158 M *t*MeCP2, with a methyl-CpG-binding domain (MBD) mutation that reduces specific DNA binding^43,44^ and is seen commonly in RTT patients.^23^ The second is P302L *t*MeCP2, which has a diminished ability to engage the TBLR1 subunit of the NCoR/SMRT repressor complex.^45^ All *t*MeCP2 variants were expressed in *E. coli*, purified to >95% homogeneity, and characterized using Western blots and LC/MS (Figure S1A,B). Variants carrying a fluorescent label were generated using sortase-A and a GGGK-lissamine rhodamine B (Rho) coreagent as previously described^9^ (Figure S1A,B). Circular dichroism (CD) analysis of all *t*MeCP2 variants confirmed that the conjugation of ZF5.3 and Tat_47–57_ had minimal effect on protein secondary structure (Figure S1C). Consistent with previous reports for full-length MeCP2,^46^ all *t*MeCP2 variants show high levels of intrinsic disorder (60%) in the absence of DNA (Table S1).

### Purified *t*MeCP2 Proteins Are Active *in Vitro*

An important function of MeCP2 is to modulate gene transcription from methylated promoters.^35^ The N-terminal methyl-CpG-binding domain (MBD) interacts with methylated DNA in the heterochromatin region^43^ while the C-terminal transcriptional-repressor domain (TRD) domain engages various partners such as the NCoR1/2 corepressor complex and CREB1.^36,45,47^ To establish whether the *t*MeCP2 proteins studied here retain these functions *in vitro*, we measured their affinities for methylated and nonmethylated DNA oligonucleotides using a fluorescence polarization assay and used immunoprecipitation methods to assess interactions with corepressor proteins in lysates (Figure 1B,C). Fluorescence polarization analysis was performed with a 22 bp double-stranded, fluorescein-tagged, DNA oligonucleotide containing a methylated or nonmethylated cytosine (Figure 1B). *t*MeCP2 interacts with methylated DNA with a *K*_D_ of 15 nM and a 15-fold preference for methylated versus nonmethylated DNA. ZF-*t*MeCP2 interacts with methylated DNA with a *K*_D_ of 55 nM and a 6-fold preference for methylated DNA. The conjugate of Tat_47–57_ and *t*MeCP2 (Tat-*t*MeCP2) interacts with both methylated DNA (*K*_D_ = 5 nM) and nonmethylated DNA (*K*_D_ = 25 nM) more favorably than *t*MeCP2 and ZF-*t*MeCP2 with a 5-fold preference for methylated DNA. As expected, ZF-*t*MeCP2(T158M) binds poorly to both methylated (*K*_D_ = 210 nM) and nonmethylated (*K*_D_ = 1.1 μM) DNA when compared to *t*MeCP2. Although the *K*_D_ describing the interaction of *t*MeCP2 with methylated DNA has not previously been determined, reported values for full-length MeCP2 fall in the range of 36–130 nM with a 2–33 fold preference for methylated DNA.^48,49^

To further probe the function of purified *t*MeCP2 proteins *in vitro*, we used an affinity pull-down assay to evaluate their interactions with the NCoR/SMRT corepressor complex in nuclear lysates of NIH3T3 cells.^28^ Lysates^47^ were incubated overnight at 4 °C with 1.5 μM of ZF-*t*MeCP2, Tat-*t*MeCP2, or *t*MeCP2; ZF-*t*MeCP2(P302L) was used as a negative control. Each *t*MeCP2 variant was extracted from the lysates using streptavidin-coated beads, and the identities and relative levels of bound NCoR/SMRT subunits (NCoR1, HDAC3, and TBL1/TBLR1) were evaluated using Western blots (Figure 1C). These blots revealed that *t*MeCP2, ZF-*t*MeCP2, and Tat-*t*MeCP2 remain intact after an overnight incubation with lysates at 4 °C and effectively sequester HDAC3, TBL1/TBLR1, NCoR1 from NIH3T3 nuclear cell lysates. In all cases, there was little or no evidence of interaction with ZF-*t*MeCP2(P302L). Taken together, these two *in vitro* assays confirm that purified ZF-*t*MeCP2 retains the core functions of MeCP2: selective recognition of methylated DNA and the ability to engage the NCoR/SMRT corepressor complex.

### Efficient Delivery of ZF-*t*MeCP2 to the Nucleus of Saos-2 and CHO-K1 Cells

**Figure 2:**
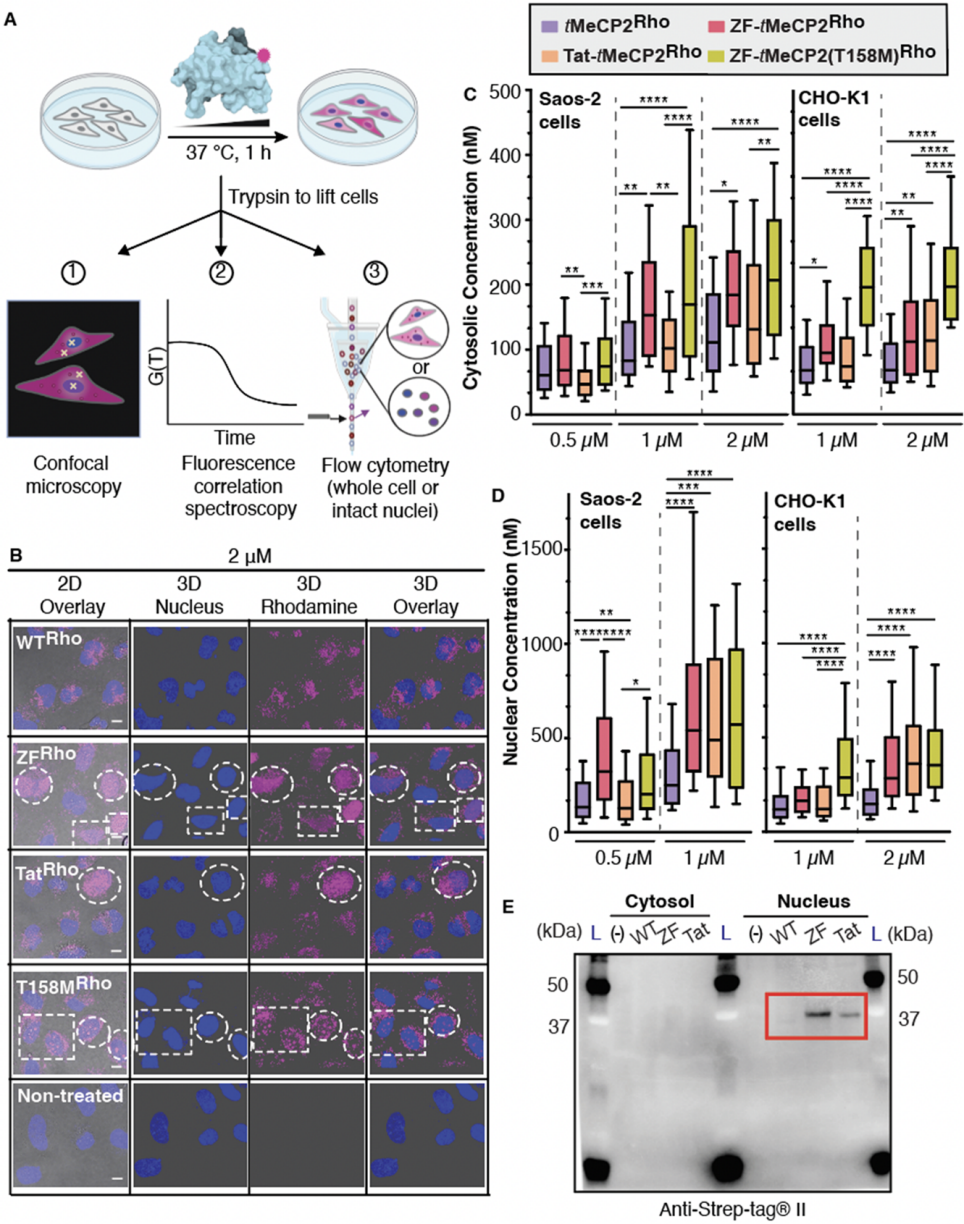
ZF5.3-*t*MeCP2 reaches the nucleus. (A) Experimental workflow. Saos-2 or CHO-K1 cells were incubated with the indicated *t*MeCP2-Rho variants for 1 h at 37 °C, 5% CO_2_; Hoechst 33342 was added during the final 5 min of the incubation period to identify the nucleus. After extensive washing and trypsin treatment to remove extracellular and surface-bound protein, cells were analyzed using 2D- and 3D-confocal microscopy and fluorescence correlation spectroscopy (FCS) or flow cytometry. (B) Representative 3D z-stacking images of Saos-2 cells treated with 2 μM of the indicated *t*MeCP2-Rho variant. Note that all proteins are labeled with Rho to a similar extent (fractional labeling between 20% and 25%). Thus, the absence of nuclear fluorescence in cells treated with *t*MeCP2-Rho is not due to differences in labeling efficiency. Cells with nuclear fluorescence are highlighted in the white dash boxes. Scale bar = 10 μm. The results shown are representative of at least two biological replicates. FCS measurements were performed on individual cells by placing the laser focus (represented by the yellow crosshairs in (A) in either the cytosol, avoiding fluorescent puncta, or the nucleus. The autocorrelation data (Figures S3–5) was fitted to a 3D anomalous diffusion equation (cytosol)^54^ or a two-component 3D diffusion equation (nucleus)^56^ using a custom MATLAB script to establish the concentration of each protein in the cytosol (C) and nucleus (D) of Saos-2 cells or CHO-K1 cells. Center line, median; box limits, 25–75 percentile; whiskers, 10–90 percentile (*n* > 30 cells total for each condition from at least two biological replicates). The intracellular concentrations of the four *t*MeCP2-Rho variants under the same treatment condition and location (e.g., 1 μM in the cytosol) were compared using Brown-Forsythe and Welch ANOVA followed by Dunnett’s T3 multiple comparisons test. ****p ≤ 0.0001, ***p ≤ 0.001,**p ≤ 0.01, *p ≤ 0.05. Each *P* value is adjusted to account for multiple comparisons. (E) Western blot analysis of the cytosolic and nuclear supernatants isolated as described in Figure S9A using an antibody against Strep-tagII (IBA 2–1509–001). Bands corresponding to intact ZF-*t*MeCP2 and Tat-*t*MeCP2 are highlighted in the red box. L, Ladder.

Next, we made use of three fluorescence-based methods and two model cell lines to evaluate the overall uptake of each *t*MeCP2 variant and specifically how much protein trafficked to the nucleus, the site of MeCP2 function. Human osteosarcoma (Saos-2) cells were incubated for 1 h with rhodamine-tagged *t*MeCP2-Rho, ZF-*t*MeCP2-Rho, Tat-*t*MeCP2-Rho, or ZF-*t*MeCP2(T158M)-Rho at concentrations between 0.5 μM and 2 μM (Figure 2A). When visualized using 2D confocal microscopy, cells treated individually with each of the four *t*MeCP2-Rho variants showed bright punctate intracellular fluorescence, while no fluorescence was observed in nontreated cells (Figure S2A). Saos-2 cells treated with ZF-*t*MeCP2-Rho, Tat-*t*MeCP2-Rho, and ZF-*t*MeCP2(T158M)-Rho also showed evidence of intranuclear fluorescence at concentrations as low as 1 μM, while cells treated *t*MeCP2-Rho did not, even at 2 μM concentration. When visualized as 3D z-stacks, cells treated with ZF-*t*MeCP2-Rho, Tat-*t*MeCP2-Rho, and ZF-*t*MeCP2(T158M)-Rho differed in intranuclear localization (Figure 2B, Figure S2B, Videos 1–4). Cells treated with ZF-*t*MeCP2-Rho and Tat-*t*MeCP2-Rho showed an even distribution of rhodamine fluorescence in Hoechst-positive, DNA-rich regions, whereas cells treated with ZF-*t*MeCP2(T158M)-Rho showed aggregated rhodamine signal in small discrete regions resembling nucleoli. This observation aligns with previous reports that truncation of the entire MBD or T158 M mutation resulted in MeCP2 relocalization to the nucleolus.^52,53^

We next used fluorescence correlation spectroscopy (FCS) to quantitatively track the cytosolic and nuclear distribution of each *t*MeCP2-Rho conjugate in Saos-2 and CHO-K1 cells (Figure 2C,D, Tables S2–3, Figures S3–7). FCS is a single-molecule technique that deconvolutes the time-dependent change in fluorescence in a small cytosolic or nuclear volume to establish both the intracellular concentration as well as the diffusion time of fluorescently labeled molecules.^54,55^ FCS analysis of Saos-2 cells revealed that all *t*MeCP2-Rho conjugates localize more significantly to the nucleus than the cytosol (Figure S6A), as established qualitatively by confocal microscopy (Figure 2B, Figure S2, Videos 1–4). Localization to the nucleus is dose-dependent between 500 nM and 1 μM, even for *t*MeCP2-Rho (Figure S6A). At low treatment concentrations (0.5 μM), the FCS-determined mean nuclear concentrations of *t*MeCP2-Rho and Tat-*t*MeCP2-Rho were lower than either ZF-*t*MeCP2-Rho (2.3-fold) or ZF-*t*MeCP2(T158M)-Rho (1.6-fold) (Figure 2D). At 1 μM, ZF-*t*MeCP2-Rho, ZF-*t*MeCP2(T158M)-Rho, and Tat-*t*MeCP2-Rho reach the nucleus more efficiently (2.0–2.3-fold) than *t*MeCP2-Rho, with localization efficiency increasing in the order *t*MeCP2-Rho < Tat-*t*MeCP2-Rho < ZF-*t*MeCP2-Rho ∼ ZF-*t*MeCP2(T158M)Rho (Figure 2D).

We note that while the conjugation of ZF5.3 to *t*MeCP2 resulted in a smaller fold-improvement in nuclear or cytosolic delivery than previously reported examples (improvements between 3^9,16^ and 32^9^ fold), the mean nuclear concentration of ZF-*t*MeCP2 established in Saos-2 cells after a 1 h incubation with 1 μM protein (709 ± 69 nM) is the highest intracellular concentration yet measured for a protein delivered by ZF5.3.^9,16^ The high concentration of ZF-*t*MeCP2 that reaches the nucleus may result from the higher intrinsic permeability of *t*MeCP2 itself in comparison to other proteins when evaluated under comparable conditions (argininosuccinate synthetase: 77 ± 30 nM, SNAP-tag: 2 ± 1 nM).^9,16^ While further studies are needed to establish the factors that lead to high intrinsic permeability, we note that *t*MeCP2 is characterized by both a high pI (10.78) and high levels (60%) of intrinsic disorder as judged by CD (Figure S1C, Table S1).

In preparation for function studies (*vide infra*), we also evaluated the trafficking of each *t*MeCP2-Rho variant to the cytosol and nucleus of CHO-K1 cells using confocal microscopy (Figure S7) and FCS (Figure 2C,D, Figure S5, Figure S6B, Table S3). These results largely mirrored the results obtained in Saos-2 cells, although overall the protein concentration in the nucleus was 1.8–3.8-fold lower than observed in Saos-2 cells. Similar cell type-to-cell type variations in delivery efficiency have been observed before among HeLa cells, SK-HEP1 cells, and Saos-2 cells.^9,16^ Nevertheless, these results provide confidence that ZF5.3 and Tat improve by 2.0–2.5-fold the nuclear delivery of *t*MeCP2-Rho in two model cell lines.

### Flow Cytometry as a High-Throughput Alternative to FCS for Evaluating Nuclear Delivery *en Masse*

The nuclear fluorescence of Saos-2 cells treated with 2 μM ZF-*t*MeCP2-Rho, Tat-*t*MeCP2-Rho, and ZF-*t*MeCP2(T158M)-Rho was too high to be reliably measured by FCS.^54,55^ Although the total fluorescence of intact cells measured using flow cytometry does not reliably quantify how much material escapes from the endosomal pathway,^9^ procedures to isolate and sort nuclei on the basis of light scattering and fluorescence are well-known.^57^ We wondered whether the fluorescence of nuclei isolated from cells treated with *t*MeCP2-Rho variants would correlate with the nuclear concentration measured in intact cells using FCS. If such a correlation was observed, then flow cytometry would provide an extremely high-throughput and rapid alternative to FCS for quantifying delivery of fluorescently tagged material to the nucleus.

To eliminate the possibility of that isolated nuclei would be contaminated by even low levels of endosomes and lysosomes, Saos-2 cells were first transduced by BacMam reagents to express Rab 5a-GFP, Rab 7a-GFP, and LAMP1-GFP (transduction efficiency = 91.6 ± 0.4%, Figure S8A). The cells were treated with 0.5–1.0 μM *t*MeCP2-Rho variants and 2 μM *t*MeCP2-Rho, and the nuclei isolated and evaluated using flow cytometry (Figure 2A). Nuclei were gated to identify the GFP negative population (59.5 ± 1.9%, Figure S8A), and on the basis of size and Hoechst 33342 fluorescence. In the concentration range evaluated, the mean nuclear fluorescence of intact Saos-2 nuclei measured by flow cytometry correlated linearly (*R*^2^ = 0.75) with the intranuclear concentrations previously measured by FCS for all four Rho-tagged *t*MeCP2 proteins, regardless of overall delivery efficiency (Figure S8B). This observation suggests that flow cytometry of intact nuclei represents a rapid alternative to FCS for high-throughput analysis of nuclear delivery. It also suggests that at a treatment concentration of 2 μM, the nuclear concentration of ZF-*t*MeCP2-Rho was above 1 μM.

To better understand the relationship between overall protein uptake and nuclear delivery, we further used flow cytometry to compare whole-cell fluorescence to that of intact isolated nuclei as a function of *t*MeCP2-Rho variant concentration and identity (Figure S8C–D). On average, the fluorescence detected in the nuclei of Saos-2 cells was 3.0–16.0-fold lower than the whole-cell fluorescence (Figure S8D). We observed a dose-dependent increase in the fraction of the *t*MeCP2-Rho variants that traveled to the nucleus. Notably, ZF-*t*MeCP2(T158M)-Rho showed consistently low nuclear localization efficiencies across all treatment concentrations, as expected since the MBD is essential for MeCP2 binding in the nucleus.^58^ Unfortunately, we could not repeat this experiment in CHO-K1 cells due to extremely low BacMam reagent transduction efficiency. We also note that although nuclear pore complex inhibitors have been reported to help to avoid false positive signals from proteins leaking into the nucleus during lysis,^58^ minimal rhodamine interference was observed in this experiment (Figure S8E).

### More ZF-*t*MeCP2 than Tat-*t*MeCP2 Remains Intact upon Reaching the Nucleus

Although fluorescence-based methods provide a reasonable initial assessment of protein delivery efficiency, they must always be accompanied by biochemical studies to ensure that the fluorescent material being followed is intact.^9,16,54^ To establish the extent to which the measured FCS values represent the concentrations of intact *t*MeCP2 proteins, we devised a workflow (Figure S9A) to stringently separate and isolate the cytosolic and nuclear fractions of Saos-2 cells after 1 h treatment with 1 μM *t*MeCP2, ZF-*t*MeCP2, or Tat-*t*MeCP2 at 37 °C. These extracts were analyzed using Western blots and an anti-Strep-tag antibody (Figure 2E). Bands corresponding to both intact ZF-*t*MeCP2 and Tat-*t*MeCP2 are evident in the nuclear supernatant. We note that although the nuclear delivery efficiencies of ZF-*t*MeCP2-Rho and Tat-*t*MeCP2-Rho determined by FCS were comparable (Figure 2D), Western blot analysis suggests that the concentration of intact ZF-*t*MeCP2 in the nucleus exceeds that of Tat-*t*MeCP2 by a significant margin. No band corresponding to *t*MeCP2 itself was observed in the nuclear supernatant, indicating that this protein was either degraded or did not enter cells at a concentration high enough to be detected. Western blot analysis of cellular extracts with antibodies recognizing highly expressed endocytic (EEA1, LAMP1, Rab7) and cytosolic (tubulin) proteins confirmed that nuclear fractions were free of detectable cytosol and endosome contaminations (Figure S9B).

To further characterize the integrity of ZF-*t*MeCP2 delivered to the nucleus, we enriched the nuclear supernatant for strep-tagged proteins, treated the enriched sample with trypsin, and subjected the digest to LC-MS/MS analysis (Figure S9C, Figure S10). More than 65% of the ZF-*t*MeCP2 sequence was detected, including fragments at the N- and C-terminus. The observation of N-terminal fragments after enrichment with a C-terminal strep tag provides further evidence that the ZF-*t*MeCP2 delivered to the nucleus is predominantly intact.

### ZF-*t*MeCP2 Accesses a HOPS-Dependent Portal for Endosomal Release

Progress in direct protein delivery has been slowed by an insufficient understanding of how non-native cargo traffics across endosomal membranes and into the cytosol. Although endosomal escape mechanisms used by certain viruses, such as influenza A,^59^ have been studied in detail, those exploited by nonviral agents–even lipid nanoparticles (LNP) used to deliver FDA-approved mRNA vaccines–remain incompletely characterized. Most improvements in delivery are empirical and modest, and the involvement of endogenous protein factors has not been rigorously excluded.

Two multisubunit tethering complexes play important roles in the endosome maturation pathway. A class C core vacuole/endosome tethering (CORVET) complex facilitates the fusion of Rab5 positive early endosomes, while the fusion of Rab7 positive late endosomes to lysosomes requires the homotypic fusion and protein sorting (HOPS)-tethering complex.^60,61^ Previous mechanistic studies indicate that efficient cytosolic and nuclear trafficking of ZF5.3 relies on the HOPS complex, but not the analogous CORVET complex.^15^ We thus sought to investigate if this dependence also held for the ZF5.3 conjugate of *t*MeCP2.

**Figure 3:**
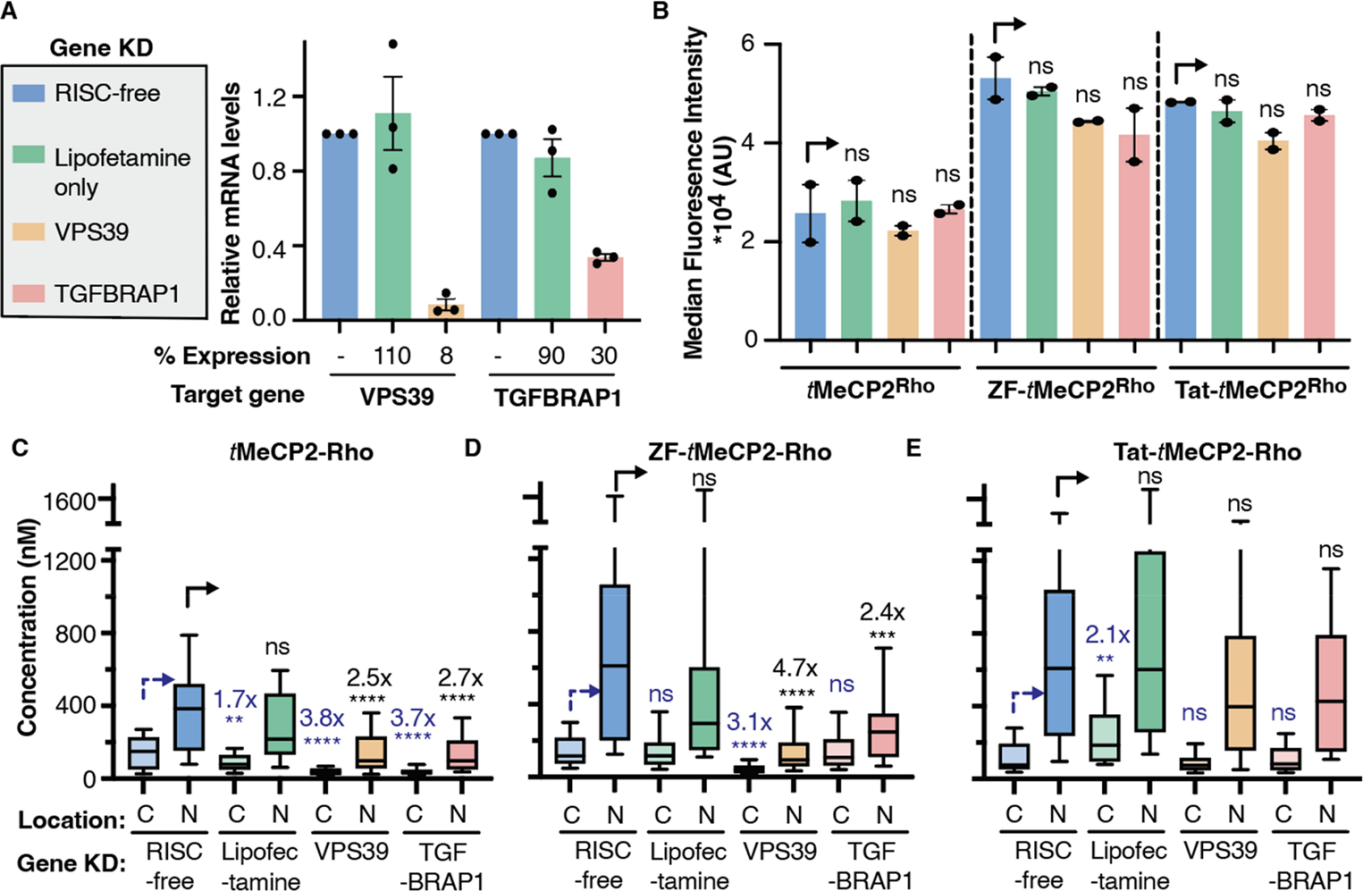
Efficient cellular trafficking of ZF-*t*MeCP2-Rho requires the HOPS complex. (A) The gene expression level of VPS39 (HOPS-specific subunits) or TGFBRAP1 (CORVET-specific subunits) in each gene knockdown sample was quantified by qPCR. Data are represented as mean ± SEM (*n* = 3). The effect of VPS39 and TGFBRAP1 knockdown on total uptake and cellular access of the indicated *t*MeCP2-Rho variant was analyzed using flow cytometry (B) and FCS (C-E), respectively. C: cytosol; N: nucleus. Center line, median; box limits, 25–75 percentile; whiskers, 10–90 percentile. For each protein treatment, the median fluorescence intensity values (B), cytosolic concentrations (C-E) or nuclear concentrations (C-E) were compared to that of a nontargeting siRNA (RISC-free) using two-tailed unpaired parametric *t* test with Welch’s correction. ****p ≤ 0.0001, ***p ≤ 0.001,**p ≤ 0.01, *p ≤ 0.05. not significant (ns) for *p* > 0.05.

We used siRNAs to knock down an essential and unique subunit of either HOPS (VPS39) or CORVET (TGFBRAP1) in Saos2 cells, using a nontargeting siRNA (RISC-free) and lipofectamine only treatment as controls (Figure 3A). The cells were then treated with 1 μM *t*MeCP2-Rho, ZF-*t*MeCP2-Rho, or Tat-*t*MeCP2-Rho for 1 h and analyzed by flow cytometry (Figure 3B) and FCS (Figure 3C–E). Total cellular uptake was affected minimally if at all by any gene knockdown (Figure 3B). Knockdown of VPS39 led to a significant decrease in the concentration of ZF-*t*MeCP2-Rho that reached the cytosol (3.1-fold) or nucleus (4.7-fold) (Figure 3D); these fold changes are consistent with those previously observed for ZF5.3 alone.^15^ Interestingly, knockdown of TGFBRAP1 also resulted in a significant (albeit smaller) decrease in the concentration of ZF-*t*MeCP2-Rho that reached the nucleus (2.4-fold). Even the trafficking of *t*MeCP2 itself was affected by the knockdown of both VPS39 and TGFBRAP1 (Figure 3C). It is well-known that depletion of TGFBRAP1 and VPS39 can affect trafficking in a cargo-dependent manner.^62^ The fact that delivery of *t*MeCP2 itself is CORVET and HOPS dependent provides one explanation for the high intrinsic permeability of this nuclear protein and deserves further study. Thus, the improved nuclear delivery of ZF-*t*MeCP2 may result because it accesses both HOPS-dependent and CORVET-dependent portals. Notably, the only *t*MeCP2 conjugate whose delivery to the cytosol and nucleus was unaffected by knockdown of either VPS39 or TGFBRAP1 was Tat-*t*MeCP2 (Figure 3E). This result suggests that Tat-*t*MeCP2 gains entry into cells, albeit less efficiently (Figure 2E), via a different subpopulation of endosomes^62^ or one or more nonendosomal pathways.^63^

We previously proposed that HOPS may engage directly with ZF5.3 to promote escape during vesicle fusion; it may also promote trafficking into intraluminal vesicles as a prerequisite to endosomal escape.^15^ The observation that the high nuclear delivery efficiency of ZF5.3-*t*MeCP2 depends on both HOPS and CORVET, favors the former explanation and deserves further study, especially as HOPS and CORVET share 4 of 6 subunits in common.^60,61^ Identification of those molecular features that promote productive interaction with HOPS and/or CORVET could improve the efficiency of other delivery strategies, including lipid nanoparticles, whose efficiency remains <10%.^64^

### Delivered ZF-*t*MeCP2 Interacts with Partner Proteins in the NCoR/SMRT Complex

**Figure 4:**
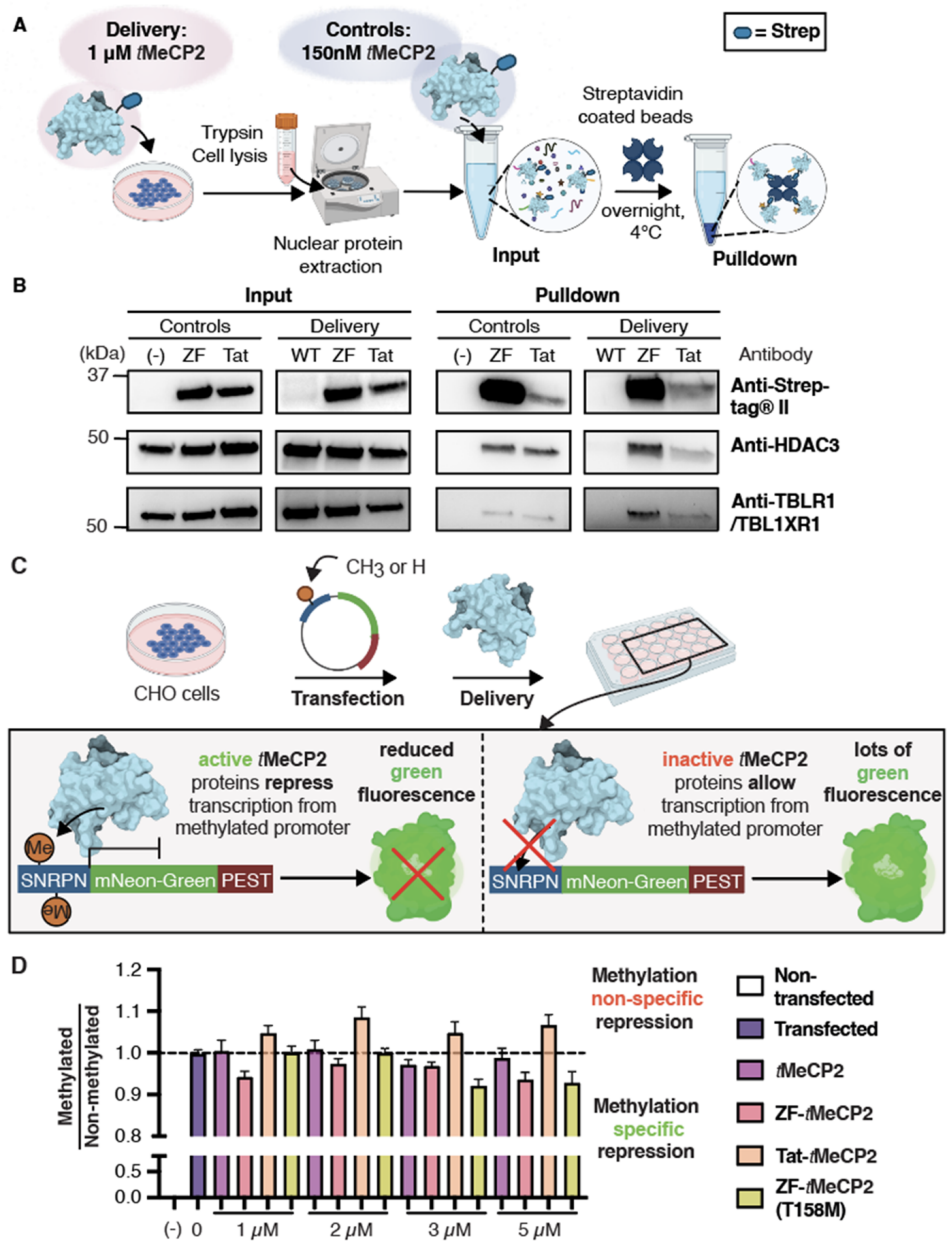
*In cellulo* assays probing *t*MeCP2 activity after delivery. (A) Co-immunoprecipitation assay workflow. CHO-K1 cells were treated with 1 μM of each *t*MeCP2 variant at 37 °C with 5% CO_2_ for 1 h. Three dishes of nontreated cells were incubated under identical conditions as controls. After incubation, cells were lifted, washed, and lysed, and the nuclear proteins were extracted (Figure S11B). The nuclear extracts of nontreated cells were doped with 150 nM ZF-*t*MeCP2 or Tat-*t*MeCP2 and then all samples were incubated with streptavidin coated magnetic beads overnight at 4 °C for the pulldown. (B) Input and pulldown samples were analyzed by Western blot using antibodies against Strep-tagII (IBA 2–1509–001), HDAC3 (CST 85057S), and TBLR1/TBL1XR1 (CST 74499S). The gel results shown are representative of two biological replicates. (C) Scheme illustrating transcription repression assay design. CHO-K1 cells were transiently transfected with a plasmid (methylated or nonmethylated) encoding mNeonGreen fluorescent protein under the control of the small nuclear ribonucleoprotein polypeptide N (SNRPN) promoter. Cells were first incubated with 1 μM *t*MeCP2 variants for 1 h at 37 °C, 5% CO_2_, and exchanged into growth media for 1 h at 37 °C, 5% CO_2_ waiting for the change in transcription to occur before they were lifted and analyzed using flow cytometry. Cells that were successfully transfected and exhibited green fluorescence higher than the background were selected to obtain the mean fluorescence intensity (MFI) under different protein treatments. The assay was performed in triplicates and more than three biological replicates were studied. (D) The selectivity of four *t*MeCP2 variants was evaluated by dividing the average normalized MFI after delivery in CHO-K1 cells transfected with methylated plasmids by that with nonmethylated plasmids. Scores are normalized against nontreated transfected cells. Data are represented as mean ± SEM. Each sample comprised 130 μL (at least 50,000 cells) and at least three technical and biological replicates at each condition.

Next we explored the function of *t*MeCP2 proteins delivered to the nucleus of CHO-K1 cells, which express low levels of endogenous MeCP2 (Figure S11A). If functional *t*MeCP2 proteins reach the nucleus, then they should interact with and sequester the essential subunits of the core NCoR/SMRT complex^28,45,47^ (NCoR1, HDAC3, and TBL1/TBLR1) upon immunoprecipitation, as observed *in vitro* in lysates (Figure 1C). To test this hypothesis, CHO-K1 cells were treated with 1 μM *t*MeCP2, ZF-*t*MeCP2, or Tat-*t*MeCP2 for 1 h at 37 °C. Nuclear proteins were rigorously isolated (Figure S11B) and incubated with streptavidin-coated magnetic beads to sequester strep-tagged *t*MeCP2 proteins and the proteins with which they interact (Figure 4A). Nontreated cells were subject to the same workflow and doped with 150 nM purified *t*MeCP2, ZF-*t*MeCP2, and Tat-*t*MeCP2. Western blot analysis confirmed that the input nuclear fractions used for immunoprecipitation were free of detectable cytosolic (tubulin) and endosomal (EEA1, Rab7, LAMP1) contaminants (Figure S11C) and contained a higher amount of intact ZF-*t*MeCP2 than Tat-*t*MeCP2 (Figure 4B), in accordance with data in Saos-2 cells in Figure 2E. Intact *t*MeCP2 could not be detected by Western blot of the nuclear extracts. Equal amounts of HDAC3 and TBLR1/TBL1XR1 were also detected in all input nuclear lysates (Figure 4B).

Examination of the Western blots after immunoprecipitation show that both ZF-*t*MeCP2 and Tat-*t*MeCP2 sequester HDAC3 and TBLR1 from nuclear lysates in accord with their effective concentration; more ZF-*t*MeCP2 reaches the nucleus intact and as a result more HDAC3 and TBLR1 are sequestered (Figure 4B). We note that the large decrease in the intensity of the Tat-*t*MeCP2 bands from input to pulldown emphasizes its sensitivity to degradation during the overnight incubation. The NcoR level was too low to be detected in CHO-K1 cells (Figure S11A). We conclude that ZF-*t*MeCP2 enters the cell nucleus and interacts more productively with partner proteins than either *t*MeCP2 or Tat-*t*MeCP2.

### Delivered ZF-tMeCP2 Selectively Represses Transcription from Methylated DNA

In the nucleus, MeCP2 acts as a bridge to deliver the NCoR/SMRT complex to methylated promoters; this recruitment represses transcription.^35^ We devised a flow cytometry assay to evaluate whether delivered *t*MeCP2 variants that reach the nucleus preferentially repress transcription of methylated over nonmethylated reporter genes (Figure 4C). Briefly, CHO-K1 cells were transfected with a methylated or nonmethylated plasmid encoding mNeonGreen fluorescent protein under the control of the small nuclear ribonucleoprotein polypeptide N (SNRPN) promoter. MeCP2 binds to the methylated form of SNRPN to downregulate downstream genes.^65,66^ A short signal sequence (PEST) was encoded at the C-terminus of mNeonGreen to promote its turnover and improve assay sensitivity.^67^ Functional *t*MeCP2 variants that reach the nucleus should selectively repress transcription from cells transfected with the methylated SNRPN promoter, leading to less mNeonGreen fluorescence relative to controls. By contrast, delivery of a trace, nonfunctional, or nonspecific *t*MeCP2 variant will either not repress transcription or do so without selectivity for the methylated promoter.

To evaluate mNeonGreen expression, CHO-K1 cells transfected with methylated or nonmethylated plasmid DNA were treated for 1 h at 37 °C with 1–5 μM *t*MeCP2, ZF-*t*MeCP2, Tat-*t*MeCP2, or ZF-*t*MeCP2(T158M) and the green fluorescence emission at 530 ± 30 nm was monitored using flow cytometry. Cells treated with *t*MeCP2 itself displayed high mNeonGreen fluorescence levels regardless of promoter methylation state (Figure S12). By contrast, cells treated with ZF-*t*MeCP2, Tat-*t*MeCP2, as well as ZF-*t*MeCP2(T158M) all showed dose-dependent decreases in mNeonGreen fluorescence. The highest levels of methylation-specific transcriptional repression was observed in cells treated with 5 μM ZF-*t*MeCP2, Tat-*t*MeCP2, as well as ZF-*t*MeCP2(T158M), although measurable effects were seen at concentrations as low as 2 μM (Figure S12A). In general, the levels of transcriptional repression were lower in cells treated with Tat-*t*MeCP2.

These data are in line with the protein nuclear concentrations (Figure 2D) and diffusion time and DNA binding kinetics measured by FCS (Table S3). FCS is useful not only for measuring the concentration of a fluorescently tagged protein or macromolecule within the cytosol or nucleus, but also for determining its intracellular diffusion time (τ_diff_).^54,55^ Fitting the autocorrelation curves obtained from intranuclear measurements in CHO-K1 cells with a two-component 3D diffusion equation^68^ identified a population of *t*MeCP2-Rho variants that diffuses freely in the nucleoplasm (fast fraction, *F*_fast_) and a second population that diffuses slowly (slow fraction, *F*_slow_), presumably because it is bound to DNA (Figure S5, Table S3). At 1 μM, the fraction of ZF-*t*MeCP2 diffuses slowly (*F*_slow_ = 26.3%) is higher than that of ZF-*t*MeCP2(T158M) (16.3%) in accord with relative methylated DNA affinities determined *in vitro* (Figure 1B) and in cells.^44^ Thus, the higher nuclear concentration of ZF-*t*MeCP2(T158M) is counterbalanced by the low DNA binding population; the result is no significant transcriptional repression (Figure S12). At 2 μM, ZF-*t*MeCP2, Tat-*t*MeCP2, as well as ZF-*t*MeCP2(T158M) all reached the nucleus at significantly higher levels than *t*MeCP2 (Figure 2D) and show higher levels of DNA binding, with values of *F*_slow_ of 24.9%, 33.7%, and 25.6%, respectively (Table S3); the result is observable transcriptional repression.

Differences in transcriptional repression are most apparent when the ratios of mNeonGreen expression in cells transfected with methylated vs nonmethylated promoters are compared (Figure 4D). As expected, no selective repression is observed in cells treated with *t*MeCP2. Tat-*t*MeCP2 treatment led to higher levels of mNeonGreen repression in cells transfected with a nonmethylated promoter, as suggested by the *in vitro* DNA binding results (Figure 1B); Tat alone possesses high nonspecific DNA binding affinity (*K*_d_ = 126 nM).^69^ The highest levels of methylation-dependent transcriptional repression were observed in cells treated with ZF-*t*MeCP2 and ZF-*t*MeCP2(T158M). As a previous study suggested, MeCP2(T158M) is capable of binding to methylated DNA in a protein level-dependent manner.^70^ At low treatment concentrations (1–2 μM), most ZF-*t*MeCP2(T158M) was sequestered in the nucleolus, so the effective nuclear concentration of ZF-*t*MeCP2(T158M) is not high enough to rescue its reduced methylated DNA binding affinity. As more protein reaches the nucleus at 5 μM treatment, a higher level of the methylated promoter is bound and repression is observed. Taken together, these data indicate that the fusion of ZF5.3 to *t*MeCP2 does not interfere with the protein’s selectivity toward methylated DNA. ZF-*t*MeCP2 reaches the nucleus at a concentration high enough to observe methylation-dependent transcription repression. Notably, although fluorescent detection implies relatively comparable nuclear delivery of ZF-*t*MeCP2 and Tat-*t*MeCP2, the latter is largely degraded (Figure 2E, Figure 4B), impairing its ability to regulate downstream transcriptions. Further study is needed to evaluate whether ZF-*t*MeCP2 also recapitulates the transcription activator activity of MeCP2.

### ZF-*t*MeCP2 Localizes to the Heterochromatin of Mouse Primary Cortical Neurons

**Figure 5:**
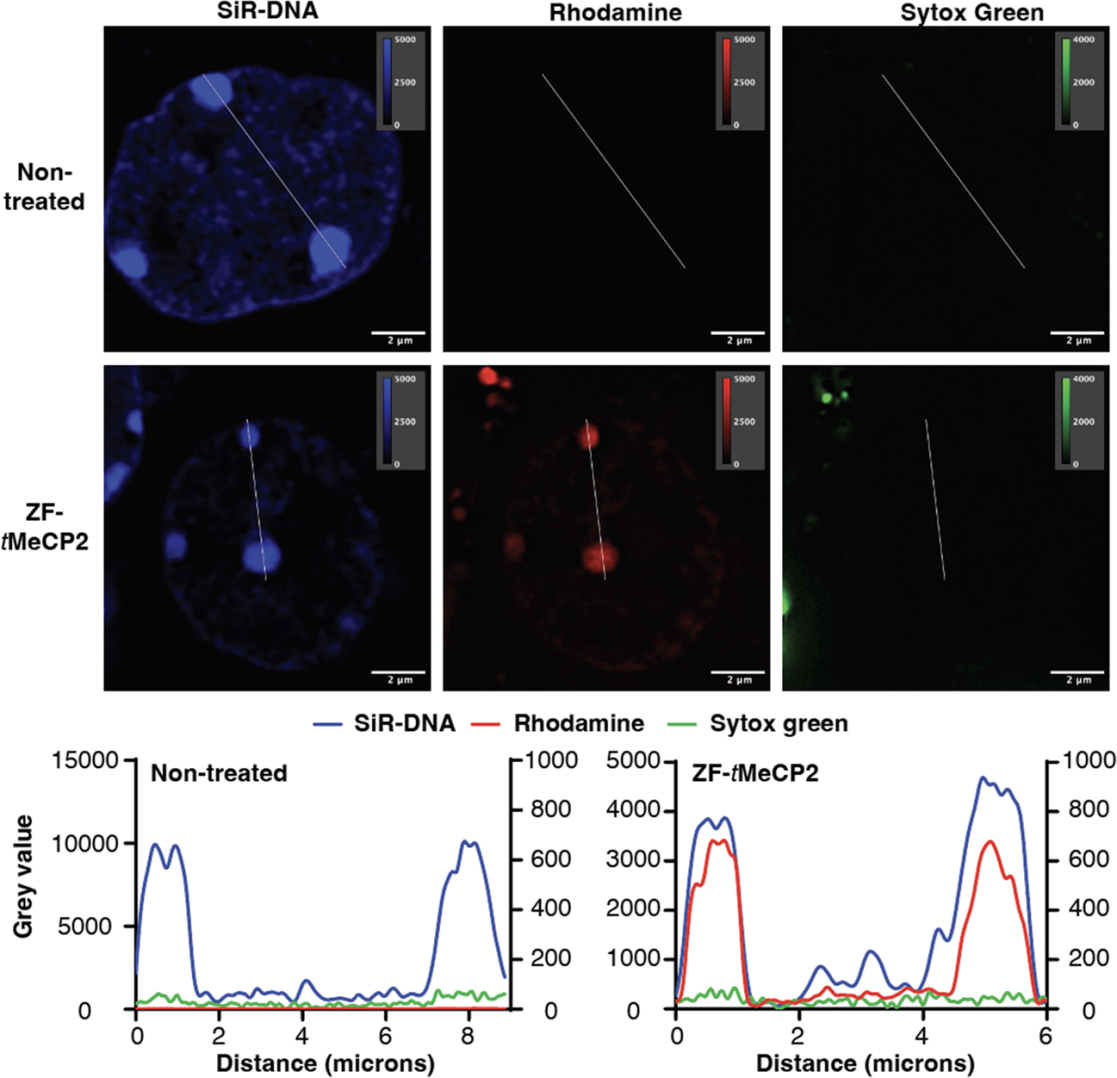
Localization of ZF-*t*MeCP2 to the heterochromatin of mouse primary cortical neurons. Cells treated with or without 1 μM ZF-*t*MeCP2-Rho for 1 h at 37 °C were imaged under a super-resolution laser-scanning microscope. The nucleus was identified using a SiR-DNA live cell nuclear stain and the SYTOX Green dye was used to exclude dead cells. Brightness and contrast of each channel were adjusted for visualization purposes, with the upper and lower thresholds indicated on each image. The pixel intensities along the indicated line within each channel image were plotted, with the SiR-DNA channel values on the left *y*-axis, Rhodamine and Sytox green channel values on the right *y*-axis for nontreated, and the SiR-DNA and Rhodamine channel values on the left *y*-axis, Sytox green channel values on the right *y*-axis for ZF-*t*MeCP2-Rho treated cells.

We further delivered ZF-*t*MeCP2-Rho to mouse primary cortical neurons, a main type of cells affected by RTT.^71^ After incubating neurons with 1 μM ZF-*t*MeCP2 for 1 h at 37 °C, we performed super-resolution imaging to evaluate the degree of neuronal delivery. While the nontreated cells showed no obvious rhodamine fluorescence, ZF-*t*MeCP2-Rho treated cells showed clear colocalization of the protein rhodamine fluorescence and SiR-DNA positive condensed chromatin (Figure 5, Figure S13), indicating efficient protein delivery and the preservation of MBD function in neuronal cells.

## Conclusion

In this work, we address the challenges of intracellular protein delivery by using the stable mini-protein ZF5.3 to hijack the endosomal maturation machinery and guide the methyl-CpG-binding-protein 2 (MeCP2) into the nucleus at a therapeutically relevant concentration. A modest dose to Saos-2 cells (1 h, 1 μM) results in a nuclear concentration of 2.1 × 10^5^ molecules/cell, which is within the range of endogenous MeCP2 in HEK293^37^ and NeuN positive neurons from mature mouse brain^19^ (1.6 × 10^5^ and 160 × 10^5^ molecules/cell, respectively). This delivered concentration is 2 orders of magnitude higher than a previously reported Tat-MeCP2 conjugate that rescued certain RTT-related symptoms.^10,39,41^ Rigorous analyses verify the absence of significant nuclear degradation and the presence of MeCP2-specific activity. Multiple independent approaches, including intact nuclear flow cytometry, a novel and general application of flow cytometry, quantitatively assess delivery in a nonamplified and high-throughput manner. Finally, the remarkable level of nuclear delivery by ZF-*t*MeCP2 enables its potential application to reverse Rett syndrome phenotypes.

## Supporting Information Available

Methods and materials, supplementary figures, supplementary tables, supplementary methods, supplementary note (PDF)
Video 1: Confocal microscopy imaging from Figure S2 (MP4)
Video 2: Confocal microscopy imaging from Figure S2 (MP4)
Video 3: Confocal microscopy imaging from Figure S2 (MP4)
Video 4: Confocal microscopy imaging from Figure S2 (MP4)
