# Supplementary material for: Dose-Dependent Nuclear Delivery and Transcriptional Repression with a Cell-Penetrant MeCP2
Source: ACS Cent Sci. 2023 Feb 3;9(2):277–88. doi: 10.1021/acscentsci.2c01226 (PMC9951310; doi:10.1021/acscentsci.2c01226)
Supplement: Supplementary file 1 — oc2c01226_si_001.pdf [file oc2c01226_si_001.pdf]

Supporting Information

Dose-dependent nuclear delivery and transcriptional repression with a cell-penetrant MeCP2

Xizi Zhang[1], Claudia Cattoglio[2, 3], Madeline Zoltek[2], Carlo Vetralla[2, 3], Deepto Mozumdar[1,4], & Alanna Schepartz[1,2,5,6*]

[1]Department of Chemistry, University of California, Berkeley, CA 94720; [2]Department of Molecular and Cellular Biology, University of California, Berkeley, CA 94720; [3]Howard Hughes Medical Institute, UC Berkeley, Berkeley, CA; [4]Department of Chemistry, Yale University, New Haven, CT 06520; [5]California Institute for Quantitative Biosciences (QB3), University of California, Berkeley, CA 94720; [6]Chan Zuckerberg Biohub, San Francisco, CA 94158

*To whom correspondence should be addressed: schepartz@berkeley.edu

Methods and Materials

Safety

No unexpected or unusually high safety hazards were encountered.

Plasmid construction

We initially attempted to express and purify full-length MeCP2 as a C-terminal fusion with ZF5.3 in *Escherichia coli*, but the yield was low and could not be optimized. The DNA sequence for *t*MeCP2(ΔNC) was obtained by deleting the N-terminal (residues 13-71) and the C-terminal (residues 313-484) sequences of mouse tMeCP2_e2 (protein sequence identifier: Q9Z2D6-1). The codon-optimized gblock encoding ZF5.3-*t*MeCP2(ΔNC)-LPETGG-Strep was purchased from Integrated DNA Technologies (IDT). A GSG linker was added between ZF5.3 and tMeCP2(ΔNC) and a GSSGSSG linker was inserted before the C-terminal LPETGG-Strep tag. A pET His6 MBP TEV LIC cloning vector (Addgene Plasmid #29656) was digested using restriction enzymes XbaI and BamHI to remove the His6-MBP-TEV portion and the synthetic gblocks containing complementary overhangs were incorporated into the pET vector using Gibson assembly. To generate *t*MeCP2(ΔNC)-LPETGG-Strep or Tat-*t*MeCP2(ΔNC)-LPETGG-Strep, the ZF5.3-*t*MeCP2(ΔNC)-LPETGG-Strep plasmid was double digested with XbaI and SacII respectively to remove the ZF5.3 segment, and ligated back using Gibson assembly with double-stranded fragments containing corresponding sequences. Point mutations (T158M, P302L) in the protein were generated by Q5 site-directed mutagenesis. The identity of all plasmids were confirmed by Sanger and whole plasmid sequencing. Relevant DNA and protein sequences are listed in **Table S5**.

Protein expression and purification

All *t*MeCP2 variants (with and without labeled fluorophore) are purified as described in **Supplementary Methods 1**.

Circular Dichroism

Circular Dichroism measurements were performed following a previously published protocol.[1] Wavelength-dependent circular dichroism spectra were recorded using an AVIV Biomedical, Inc. (Lakewood, NJ) Circular Dichroism Spectrometer Model 410 at 25 °C in a 0.1 cm pathlength quartz cuvette. CD spectra were collected at ~12 µM protein concentration in 20 mM HEPES, 300 mM NaCl, 10 % glycerol pH 7.6 between 300 and 200 nm at 1 nm intervals with an averaging time of 5 seconds. A separate wavelength spectrum for storage buffer alone was performed to verify no interfering buffer signal. Raw ellipticity values were converted to mean residue ellipticity using the equation

$$\theta = \frac{m^\circ \cdot MRW}{l \cdot C}$$

where θ = mean residue ellipticity in deg·cm^2 ·dmol^{-1}, $m°$ = raw ellipticity values in millidegrees, *MRW* = mean residue weight (calculated as the molecular weight divided by the number of backbone amides), l = path length of the cuvette in millimeters, and C = concentration of protein in mg mL^{-1}. The protein secondary structure was predicted using the webserver BeStSel (http://bestsel.elte.hu/index.php).

Fluorescence polarization

Samples for fluorescence polarization (FP) studies were prepared by mixing assay buffer (25 mM Tris-HCl, 6% glycerol, 100 µg/mL BSA, 0.1 mM EDTA, and 0.1 mM TCEP, 250 mM KCl, pH 7.6), different *t*MeCP2 variants with final concentrations ranging from 1 nM to 5 µM and 20 nM DNA probe. The DNA probes carrying an N terminal 6-fluorescein[2] (methylated or non-methylated, **Table S5**) were synthesized by annealing equimolar of complementary single strands derived from a 22 bp DNA segment of mouse BDNF promoter IV (purchased from IDT) at 95 °C for 2 min, followed by 60 °C for 10 min and cooled to 4 °C.[3] The samples were incubated for 30 min at room temperature to reach equilibrium and added to a 96 well half area solid black plate (Corning #3993, 50 µL per well).

FP measurements were performed using a SpectraMax M5 plate reader with an excitation wavelength at 480 nm and an emission window of 515 to 520 nm. Measurements from three wells were averaged for each determination. To calculate K_D, the data were fitted by least squares regression to an equilibrium binding equation based on the Langmuir model with modifications explicitly considering the total DNA concentration[4] using GraphPad Prism 9**.**

$$F_{obs} = F_0 + \frac{(F_{max} - F_0)(A - \sqrt{A^2 - 4LP^n})}{2L}$$

where $A = P^n + L + K_D^n$. In this equation F_0 is the background FP value of the DNA probe when no protein was added; F_{obs} is the FP detected by the plate reader for each sample; F_{max} is the maximum FP at saturation; P and L represent the total protein and DNA concentration, respectively; K_D is the equilibrium dissociation constant; and n is the Hill coefficient. While previous studies reported both MBD[4] and full-length tMeCP2[5,6] can bind cooperatively to DNA, the high salt concentration used in the assay buffer suppressed binding cooperativity and preserved the specificity for methylated DNA.[6] The Hill coefficients fitted for all conditions in this experiment were close to 1.

Cell culture

Saos-2 cell stock was purchased from the American Type Culture Collection (ATCC). NIH3T3, HeLa and CHO-K1 cell stocks were purchased from UC Berkeley Cell Culture Facility. Saos-2 cells were cultured in McCoy's 5A medium (Hyclone) with phenol red containing 15% fetal bovine serum (FBS), penicillin and streptomycin (P/S, 100 units/mL and 100 µg/mL respectively), 1mM sodium pyruvate (Gibco), 2 mM GlutaMax (Gibco). NIH3T3 and HeLa cells were cultured in Dulbecco's Modified Eagle's Medium (DMEM, Gibco) with 10% FBS and P/S.

CHO-K1 cells were cultured in F12 Nutrient Mixture (Ham) media (Gibco) with L-Glutamine, 10 % FBS and P/S. All cell cultures were incubated at 37 °C, in 5% CO_2.

***In vitro* pull-down assay**

Nuclear lysate of NIH3T3 cells were obtained as described in **Supplementary Methods 2**. 200 µL of the nuclear lysate was mixed with 1.5 µM of purified *t*MeCP2 variants overnight at 4℃ in the coIP buffer[7] (20 mM HEPES, pH 7.6, 10 mM KCl, 150 mM NaCl, 1 mM $MgCl_2$, 0.1% Triton X-100 (vol/vol), protease inhibitors (Roche), 15 mM BME). On the next day, to pull down *t*MeCP2 protein complexes, MagStrep "type3" XT magnetic beads (IBA 2-4090-002) following the manufacturer's note were used. The beads were pre-equilibrated with coIP buffer and incubated with the mixture of nuclear lysate and *t*MeCP2 for 45 min at RT on a rotating wheel. After separating the beads from the unbound supernatant using a magnetic separator, the beads were washed with 200 µL coIP buffer two times (quickly vortex and centrifuge). The pull-down proteins were eluted with 10 µL 5x SDS-PAGE loading dye and 10 µL milliQ water at 95 ℃ for 3 min. The input and pulldown samples were analyzed by western blot using primary antibodies against Strep-tagII (IBA 2-1509-001), HDAC3 (CST 85057S), TBLR1/TBL1XR1 (Abcam ab190796), NCoR1 (CST 5948S), and secondary antibody HRP-linked Anti-rabbit IgG (CST 7074S).

Fluorescence-based assay for protein delivery

The fluorescence-based experiments (confocal microscopy, fluorescence correlation spectroscopy) to evaluate protein delivery were performed according to previously published protocols.[8] For delivery into Saos-2 cells, 500 µL *t*MeCP2 variants diluted in McCoy's 5A medium (–FBS, –phenol red) to final concentrations (0.5 - 2 µM) were added to the cells plated in a 6-well plate to incubate for 1h at 37°C, 5% CO_2. 300 nM of the nuclear stain Hoechst 33342 was added to cells 5 min prior to the end of incubation time.

For experiments using CHO-K1 cells, a similar protocol was followed except: the cells were plated in DMEM media supplemented with 1x non-essential amino acid (NEAA) +10% FBS, without phenol red on day 1. On day 2, 1-5 µM *t*MeCP2 variants were diluted in DMEM media supplemented with 1x NEAA (-phenol red) for delivery. DMEM media supplemented with 1x NEAA (+10% FBS, -phenol red) was used to quench the trypsin reaction and rinse the 6-well plate. Cells were resuspended in DMEM media supplemented with 1x NEAA (–phenol red) for confocal microscopy and fluorescence correlation spectroscopy studies.

Confocal Microscopy and Fluorescence Correlation Spectroscopy (FCS)

Confocal microscopy and FCS experiments were performed using a STELLARIS 8 confocal microscope (Leica) with a Hybrid HyD X detector, and an HC PL APO 63x/1,20 W motCORR CS2 water (63W) immersion objective (Leica). The correction collar of the objective was adjusted based on the thickness of an 8-well chambered coverglass prior to experiments. The fluorophore was excited at 561 nm and the fluorescence filter was set to 570-660nm. The pinhole

of the 561 nm laser was set to 1 AU. At the beginning of the experiments, the imaging chamber was equilibrated to 37°C, 5% CO_2. Before each FCS measurement, a confocal image of the cells was obtained and the laser was directed to either the cytosol or nucleus of the cells to obtain FCS data in discrete cellular locations. Areas around the punctate fluorescence of endosomes were avoided. All FCS measurements were collected in ten consecutive five-second time intervals. A minimum of 20 cells was measured per replicate, and data from at least two biological replicates were collected for each condition. To measure the diffusion coefficients of *t*MeCP2-Rho variants *in vitro*, proteins were diluted in DMEM (25 mM HEPES, -phenol red) to 100 nM fluorophore concentration, and their autocorrelation data were collected at 37°C (10 repeats, 5s intervals). Because some phototoxicity was observed during FCS in CHO-K1 cells, besides keeping the cells at 37°C, 5% CO_2, we avoided shining light on a single cell two times by measuring the cytosol, and nuclear concentrations in separate cells.

The cytosolic and nuclear concentrations of fluorescently tagged *t*MeCP2-Rho variants in cells were calculated by fitting the measured data to a 3D anomalous diffusion equation (cytosol) and a two-component 3D diffusion equation (nucleus). We further adjusted the calculated concentrations based on the fluorophore labeling efficiency of each protein to obtain the true intracellular concentrations (**Supplementary Note 1**).

Flow cytometry

Saos-2 cells (0.1 million cells per well in 667 µL McCoy's 5A + 15% FBS - phenol red media) in a 24 well plate were transfected with endosomal and lysosomal markers 20 hr before experiment. For transfection, CellLight™ Early Endosomes-GFP, BacMam 2.0 (Thermo Fisher, C10586) and CellLight™ Late Endosomes-GFP, BacMam 2.0 (Thermo Fisher, C10588) were added at MOI of 10. CellLight™ Lysosome-GFP, BacMam 2.0 (Thermo Fisher, C10596) was added at MOI of 5. After protein delivery (170 µL per well), cells were washed and trypsinized as described above. After centrifugation at 200 g for 3 min, the pellet was resuspended in 1 mL DPBS and 100 µL was saved for whole-cell flow cytometry analysis. The rest of 900 µL was transferred to a 1.5 mL microcentrifuge tube and pelleted at 500 g for 3min. The pellet was lysed with precooled 500 µL lysis buffer (290 mM sucrose, 10 mM imidazole, pH 7.0, 1 mM DTT, 0.02% NP-40) and centrifuged at 1200 g, 4 °C for 7 min. The pellet was resuspended again in 200 µL of lysis buffer, centrifuged at 1200 g, 4 °C for 7 min, and repeated. The final resulting intact nuclei pellet was resuspended in 200 µL PBS. Flow cytometry measurements were performed at room temperature with an Attune NxT flow cytometer. Hoechst 33342 excited with a laser at 405 nm, and the emission filter was set at 440 ± 50 nm. The fluorophore Rho was excited with a laser at 561 nm, and the emission filter was set at 585 ± 16 nm. GFP was excited with a laser at 488 nm, and the emission filter was set at 530 ± 30 nm. The collected data were analyzed using FlowJo software (version 10.8.1, FlowJo, LLC).

RNAi

siRNA-mediated knockdown was performed as described previously[9] using Lipofectamine RNAiMAX transfection reagent (Invitrogen) and siRNA (100 nM, Dharmacon). All siRNA used was listed in **Table S5**. Saos-2 cells were transfected for 4 hours and grew in McCoy's 5A medium (2 mL/well, 15% FBS, 1 mM sodium pyruvate, 2 mM GlutaMax, -phenol red, -P/S) for 72 h at 37 °C, 5% CO2 for optimal gene knockdown. The effect of gene knockdown was studied using flow cytometry and FCS as described above.

RT-qPCR

The degree of siRNA-mediated gene knockdown after 72 h was evaluated using RT-qPCR as described previously.[9] For RT-qPCR, cDNA was amplified using 150 nM gene-specific primers (IDT, PrimeTime qPCR primers) with SsoFast EvaGreen Supermix (Bio-Rad) on a Bio-Rad CFX96 real-time PCR detection system. Three biological replicates were evaluated. Each run includes the siRNA-targeted gene, GAPDH as the reference gene and their respective negative controls (no reverse transcriptase and no template). Each sample was run in triplicate. Primers used for reverse transcription into cDNA and qPCR were listed in **Table S5**.

Cytosolic fractionation to determine protein intactness

The cellular fractionation experiment was adapted from previous reports[9–11] with modifications to ensure pure nuclei isolation. Saos-2 cells were grown to ~2.5×10^6 cells in a 100 mm dish in McCoy's 5A medium (+15% FBS, -phenol red). For protein delivery, *t*MeCP2-Strep variants diluted in clear McCoy's media without FBS to final concentration of 1 µM were incubated with cells at 37 °C with 5% CO_2 for 1 h. One dish of cells with the same clear McCoy's media added was included as a non-treated control. After incubation, cells were lifted, washed, and lysed as previously described.[11] The homogenized cell lysate was centrifuged for 10 min at 800 g, 4 °C. The crude supernatant was re-centrifuged at 1200 g, 4 °C for 10 min. The resulting supernatant was transferred to a polycarbonate ultracentrifuge tube and centrifuged at 350 kg for 30 min (TL-100; Beckman Coulter, TLA-100 rotor (20 x 0.2 mL) to isolate the cytosolic fraction. The crude nuclei pellet was washed in 500 µL of isotonic sucrose buffer (290 mM sucrose, 10 mM imidazole, pH 7.0, 1 mM DTT, and 1 cOmplete protease inhibitor cocktail (Roche)) supplemented with 0.15% NP-40. After incubating on ice for 10 min, the mixture was centrifuged at 1200 g, 4 °C for 10 min. The resulting pure nuclear pellet was first resuspended with 20 µL milliQ water and 2 µL benzonase (Sigma, 71206) at room temperature for 20 min. 80 µL high salt extraction buffer[12] (20 mM HEPES, pH 7.6, 1.5 mM $MgCl_2$, 420 mM NaCl, 0.2 mM EDTA, 25% glycerol, protease inhibitor (Roche)) was then added to the mixture and vortexed vigorously during the 30 min incubation period on ice. The extracted nuclear supernatant was obtained by centrifuging at 21,000 g, 4 °C for 5 min. For SDS-PAGE gel, both the cytosolic pellet and the nuclear pellet were dissolved in 20 µL of 5x SDS gel loading dye and boiled at 95 °C for 5 min. All the supernatant samples (cytosolic, wash, nuclear) were prepared by mixing 20 µL sample with 5 µL 5x SDS gel loading dye and boiled at 95 °C for 5 min. Loading controls were generated by adding 150 nM of purified *t*MeCP2-Strep variants to non-treated nuclear supernatant. Western blot analysis was performed using primary antibodies

against Strep-tagII (IBA 2-1509-001), MeCP2 (CST 3456S), LAMP 1 (CST 9091S), EEA1 (CST 3288S), tubulin (CST 2125S), Rab 7 (CST 9367S) and the secondary antibody HRP-linked Anti-rabbit IgG (CST 7074S).

LC-MS/MS analysis

1 µM ZF-*t*MeCP2-Strep was incubated with Saos-2 cells (37 °C, 5% CO_2, 1 h) and the nuclear supernatant was isolated as described in the section above. ZF-*t*MeCP2-Strep delivered to the nucleus was enriched by incubating the nuclear supernatant with MagStrep "type3" XT magnetic beads (IBA 2-4090-002) for 45 min at RT in a rotating wheel. After separating the beads from the unbound supernatant using a magnetic separator, the pull-down proteins were eluted with 10 µL 5x SDS-PAGE loading dye (diluted to 2.5x by 10 µL milliQ water) at 95 °C for 3 min. The sample was run on a 10% SDS-PAGE gel (Biorad, Mini-PROTEAN® TGX™) and visualized by Gelcode Blue® Coomassie stain (Pierce) following the manufacturer's note. The band corresponding to ZF-*t*MeCP2-Strep was extracted from the gel and digested with trypsin, and the resulting peptides were dried and resuspended in buffer: 5% acetonitrile/ 0.02% heptaflurobutyric acid (HBFA)). Mass spectrometry was performed by the Proteomics/Mass Spectrometry Laboratory at UC Berkeley as described in **Supplementary Methods 3**.

Determining endogenous level of MeCP2 and NCoR

1×10^6 of Saos-2, CHO-K1, NIH3T3 or HeLa cells were lysed with 100 µL M-PER™ Mammalian Protein Extraction Reagent (Thermo Scientific, 78501) for 10 min following the manufacturer's instructions. The lysates were centrifuged at 14,000 g for 15 min. For SDS-PAGE gel, the pellets were dissolved in 20 µL of 8 M urea and 5 µL 5x SDS gel loading dye and boiled at 95 °C for 5 min. The supernatant samples were prepared by mixing 20 µL sample with 5 µL 5x SDS gel loading dye and boiled at 95 °C for 5 min. Western blot analysis was performed using primary antibodies against MeCP2 (3456S), NCoR1 (CST 5948S), GAPDH (CST 2118S), tubulin (CST 2125S) and the secondary antibody HRP-linked Anti-rabbit IgG (CST 7074S).

***In cellulo* coimmunoprecipitation assay**

On day 1, ~5×10^6 CHO-K1 cells were plated in 150 mm dishes in F12 Nutrient Mixture (Ham) media with L-Glutamine (+10 % FBS). On day 2, for protein delivery, cells were washed with 25 mL DPBS three times and treated with 1 µM tMeCP2-Strep variants (WT, ZF or Tat, diluted in F12 media without FBS) at 37 °C with 5% CO_2 for 1 h. Three dishes of cells were incubated with the same F12 media added as controls. After incubation, cells were lifted, washed and lysed as described in the cellular fractionation section. For lysis, the cells from each dish were then suspended in 300 µL of isotonic sucrose buffer (290 mM sucrose, 10 mM imidazole, pH 7.0, 1 mM DTT, and 1 cOmplete protease inhibitor cocktail (Roche)), transferred to 0.5 mL microtubes containing 1.4 mm ceramic beads (Omni International) and homogenized using a Bead Ruptor 4 (Omni International) for 10 s at speed 1. The homogenized cell lysate was centrifuged for 10 min at 800 g, 4 °C. The supernatant contained cytosolic proteins and the crude nuclei pellet was washed in 500 µL of isotonic sucrose buffer supplemented with 0.15% NP-40. After incubating

on ice for 10 min, the mixture was centrifuged at 1200 g, 4 °C for 10 min. After separating the wash 1 supernatant, the pellet was washed again with 400 µL of isotonic sucrose buffer supplemented with 0.15% NP-40 and centrifuged at 1200 g, 4 °C for 10 min. The resulting pure nuclear pellet was first resuspended with 70 µL low salt buffer (20 mM HEPES, pH 7.6, 1.5 mM $MgCl_2$, 10 mM KCl, 25% glycerol, protease inhibitor (Roche)) and 2.5 µL benzonase (Sigma, 71206), transferred to 0.5 mL microtubes containing 1.4 mm ceramic beads and homogenized using a Bead Ruptor 4 for 10 s at speed 5 for three times (30 s interval on ice between each session). The homogenized nuclear lysate was then incubated at 37 °C for 10 min on a rotating wheel for benzonase digestion. 1M NaCl solution was added to the lysate to a final NaCl concentration of 430 mM. The mixture was homogenized again for 10 s at speed 5 and incubated for 1 h at 4 °C on a rotating wheel. The extracted nuclear supernatant was obtained by centrifuging at 18,000 g, 4 °C for 10 min.

For two control samples, 150 nM purified ZF- or Tat-*t*MeCP2-Strep was doped in nuclear supernatant. The input samples were prepared by taking 12.5 µL of nuclear supernatant and mixing with 7.5 µL milliQ water and 5 µL 5x SDS gel loading dye. The rest of the nuclear supernatant was diluted to 150 mM NaCl with low salt buffer supplemented with 15 mM BME and incubated with MagStrep "type3" XT magnetic beads (IBA 2-4090-002) overnight at 4 °C on a rotating wheel. On the next day, the unbound supernatant was separated from the beads using a magnetic separator. The beads were washed with 200 µL coIP buffer two times (quickly vortex and centrifuge) and eluted with 10 µL 5x SDS-PAGE loading dye (diluted to 2.5x by 10 µL milliQ water) at 95 ℃ for 3 min. The input and pulldown samples were analyzed by western blot using primary antibodies against Strep-tagII (IBA 2-1509-001), HDAC3 (CST 85057S), TBLR1/TBL1XR1 (CST 74499S), and secondary antibody HRP-linked Anti-rabbit IgG (CST 7074S). To detect for contamination from other cellular compartments, the input and pulldown samples, as well as other fractions during the isolation process (cytosolic supernatant, wash 1, and wash 2), were analyzed by western blot using primary antibodies against EEA1 (CST 3288S), tubulin (CST 2125S), Rab 7 (CST 9367S) and the secondary antibody HRP-linked Anti-rabbit IgG (CST 7074S).

***In cellulo* transcription assay**

To construct the reporter plasmid, the mNeonGreen gene was amplified from a pCS2+mNeonGreen-C Cloning Vector (Addgene #248605) using design primers (**Table S5**). The SNRPN promoter was amplified from mouse genomic DNA (Promega, G3091) using design primers (**Table S5**). The amplified SNRPN promoter segment was incorporated into a mammalian expression vector pNL1.2 (Promega, N1011) using the two restriction sites NheI and HindIII followed by gibson assembly. The assembled pNL1.2-SNRPN plasmid was confirmed by Sanger sequencing and digested again with HindIII and EcoRI to incorporate the amplified mNeonGreen gene downstream of SNRPN using a gibson assembly kit. The final assembled plasmid was named as pSNRPN-mNeonGreen-PEST. To generate methylated promoter, the plasmid was with HpaII methyltransferase (NEB, M0214S) following manufacturer’s

instructions. Complete methylation was checked by testing the plasmid's resistance to HpaII restriction enzyme digestion.

To perform the assay, on day 1 CHO-K1 cells grown to ~70% confluency in 60 mm dishes were transfected with either methylated or non-methyalted reporter plasmids in reduced serum media (OPTI-MEM, Gibco, 31985-062) using a TransIT CHO-K1 transfection kit (Mirusb Bio) following manufacturer's instructions. After 12 h the cells were washed with DPBS two times and lifted with enzyme-free cell dissociation buffer (Gibco). The lifted cells were pelleted, counted and plated into a 24-well plate in 1.2×10^5 cells/well in growth media (F12 Nutrient Mixture (Ham) media (Gibco) with L-Glutamine + 10 % FBS). On day 2, the medium was aspirated and the cells were washed with 1 mL DPBS two times. Then 130 µL of 1-5 µM *t*MeCP2-Strep variants diluted in F12 medium (-FBS, -P/S) were added to the cells and incubated for 1 h at 37 °C, 5 % CO_2. After delivery, cells were washed with 1 mL of DBPS three times and the media was replaced with the growth media for a one-hour incubation. After incubation, the cells were lifted with 200 µL TrypinLE Express for 5 min at 37 °C, 5% CO_2, quenched with 1 mL growth media, and transferred to 1.5 mL microcentrifuge tubes. The cells were pelleted by centrifugation at 500 g for 3 min, and washed with 1 mL clear DMEM medium. After centrifuging at 500 g for 3 min, the pellets were resuspended in 200 µL clear DMEM medium and analyzed using an Attune NxT flow cytometer (Life Technologies).

For flow cytometry, the mNeonGreen protein was excited with a laser at 488 nm, and the emission filter was set at 530 ± 30 nm. 130 µL (at least 50,000 cells) were analyzed for each sample and at least three technical and biological replicates were measured for each condition. Cells having green fluorescence were gated based on the background fluorescence level of cells not transfected with reporter plasmids.

Animals

All animal experiments were performed in strict accordance with the recommendations in the guide for the care and use of laboratory animals of the National Institutes of Health and following the animal use protocol AUP-2015-09-7988-2 approved by the Animal Care and Use Committee (ACUC) of the University of California, Berkeley. C57BL/6 mice were mated and newborn pups (postnatal day 1, P1) sacrificed by decapitation. Brains were immediately extracted and dissected to isolate cortical neurons (**Supplementary Methods 4**).

Staining of Primary Cortical Neurons

Before staining, we removed the CNB media from cells, filtered it (0.22 µm) and used it as conditioned media during the following incubation steps. We then incubated neurons in conditioned CNB containing 500 nM SiR-DNA live cell nuclear stain (Cytoskeleton, CY-SC00) for an hour in a regular cell incubator. Cells were washed twice in equilibrated CNB and either left untreated or treated with 1 µM ZF-tMeCP2-Rho protein in conditioned CNB for an hour in a regular cell incubator. Cells were washed again twice and incubated in equilibrated CNB

containing 1 µM SYTOX® Green dye (ThermoFisher Scientific, S7020) for 10 minutes in a regular cell incubator. After 3 final washes cells were added of conditioned CNB and subject to super-resolution imaging.

Live Cell Confocal imaging of Primary Cortical Neurons

Super-resolution imaging of live cortical neurons was performed on a Zeiss LSM 900 laser-scanning microscope with an inverted Axio Observer equipped with an Airyscan 2 detector, a motorized stage and an incubation chamber maintaining the sample at 37 °C and 5% CO_2, operated by the ZEN 3.1 blue edition software. Images were acquired using a 63x Plan-Apochromat NA1.4 oil-immersion objective at an 8x zoom, corresponding to a 20-nm xy pixel size. The SYTOX® Green dye was excited with 488 nm (diode laser set at 0.1% with a master gain of 800 V), rhodamine was excited with 561 nm (diode laser set at 0.2% with a master gain of 850 V) and SiR-DNA was excited with 640 nm (diode laser set at 0.75% with a master gain of 850 V). The three channels were acquired sequentially. Live cells were selected based on exclusion of the SYTOX® Green dye, which only penetrates compromised plasma membranes. Detection was performed on the Airy disk consisting of 32 GaAsP photomultiplier tube detectors with a detector range of 497-550 nm for the 488-nm laser, 566-630 nm for the 561-nm laser and 645-700 nm for the 640-nm laser. For each cell and channel, 21 to 27 z-planes were acquired with a z interval of 150 nm. Z-stacks were Airyscan processed in 3D using the ZEN 3.1 blue edition software with autofiltering. Colocalization analysis between the rhodamine and the SiR-DNA channels was performed in ImageJ/Fiji2. Brightness and contrast of each channel was adjusted for visualization purposes, with the upper and lower thresholds indicated on each image.

Statistical tests

All statistical tests were done using GraphPad Prism 9 software. Statistical significance for flow cytometry and FCS data were assessed by Brown-Forsythe and Welch ANOVA followed by Dunnett's T3 multiple comparisons test or unpaired t test with Welch's correction. The values in the transcriptional repression assay were compared using nonparametric Kruskal-Wallis multiple comparison test. The test used and error bars are defined in each figure legend.

Data availability. The data underlying this study are not publicly available due to patient privacy issues. The data are available from the corresponding author upon reasonable request. The MATLAB® script used to analyze FCS measurements is available from GitHub (https://github.com/schepartzlab/FCS).

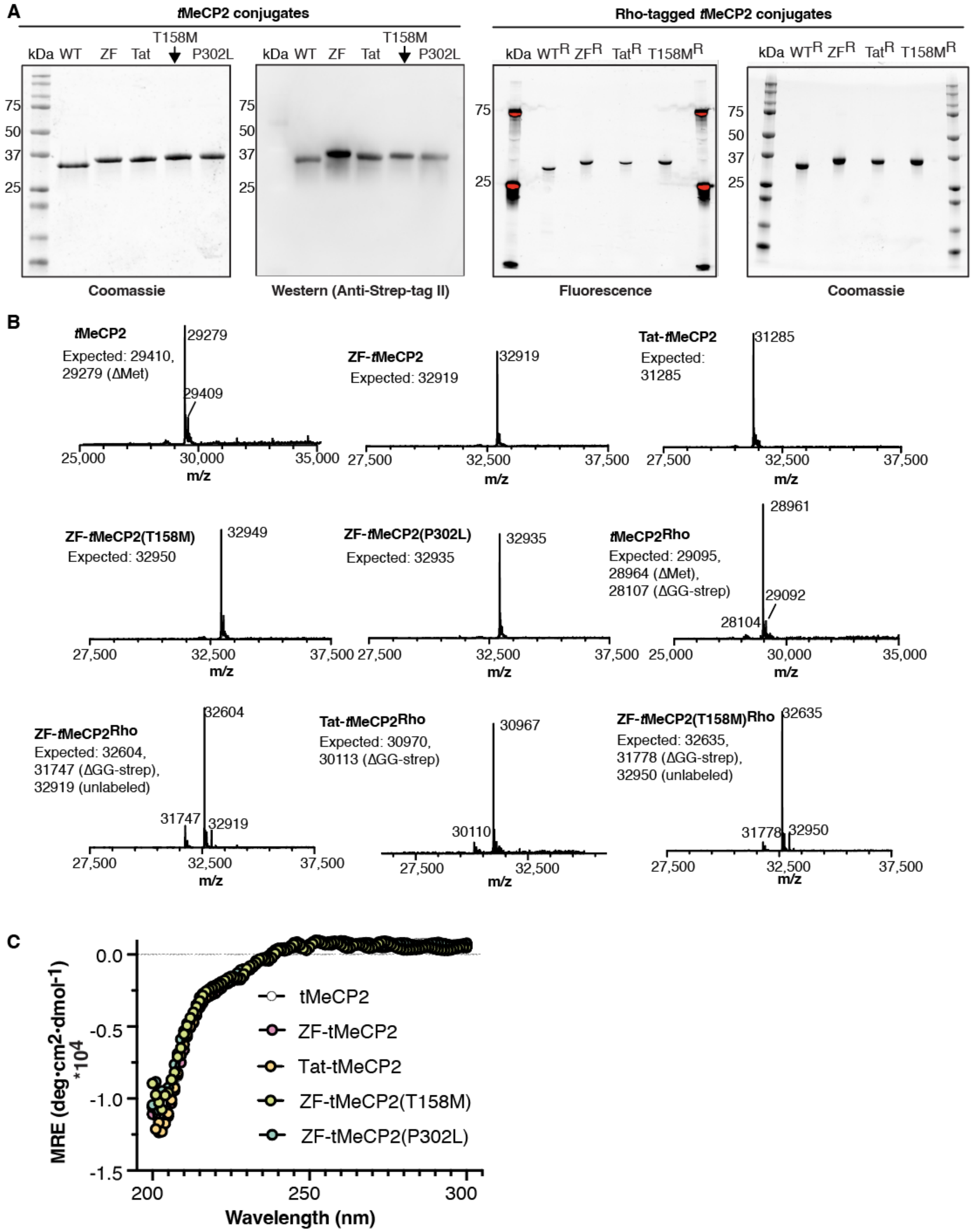

Figure S1. **Characterization of purified *t*MeCP2 proteins using SDS-PAGE and mass spectrometry.** (A) (left) Coomassie-stained SDS-PAGE gel and western blot showing the five final purified *t*MeCP2 variants studied herein. (right) Fluorescence imaging confirmed

conjugation of Rho to the indicated fusion protein. Labeling efficiencies range from 48-67%. SDS-PAGE shows that purified fusion proteins are all > 90% homogeneous. (B) LC/MS analysis confirmed the identity of the indicated fusion protein. Prominent peaks were observed for the labeled protein, as well as the presence of unlabeled protein and an intermediate product from sortase labeling. (C) CD spectra of *t*MeCP2 fusion proteins were collected at ~12 µM protein concentration in 20 mM HEPES, 300 mM NaCl, 10% glycerol pH 7.6 at RT between 200 and 300 nm at 1 nm intervals with an averaging time of 5 seconds. The protein secondary structure was predicted (**Table S1**) using the webserver BeStSel (http://bestsel.elte.hu/index.php).

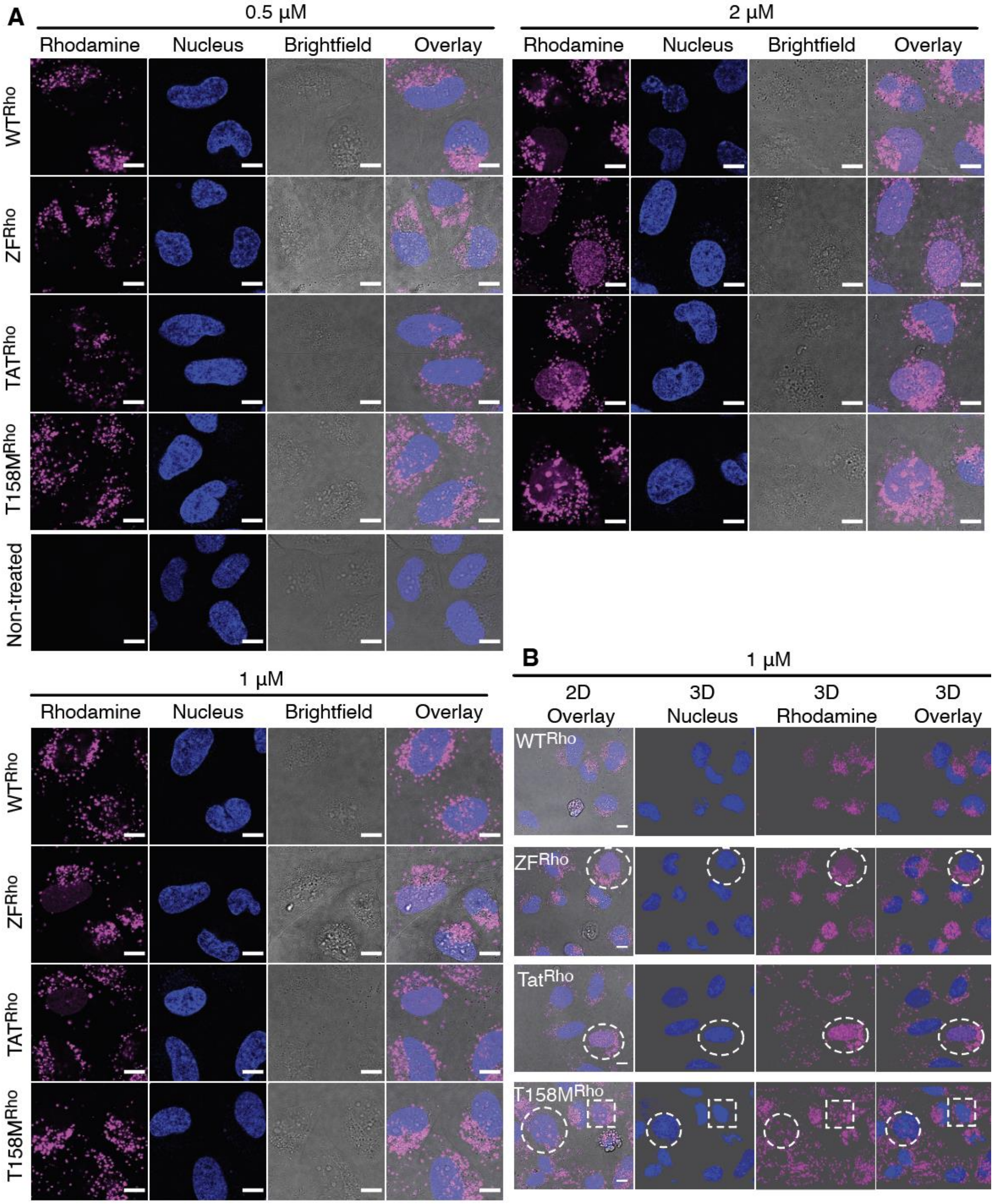

Figure S2. Confocal microscopy images of Saos-2 cells treated with *t*MeCP2-Rho variants. Representative 2D (A) and 3D (B) confocal images of Saos-2 cells treated with different concentrations of *t*MeCP2-Rho variants (WT^{Rho}: *t*MeCP2^{Rho}, ZF^{Rho}: ZF-*t*MeCP2^{Rho}, Tat^{Rho}: Tat-*t*MeCP2^{Rho}, T158M^{Rho}: ZF-*t*MeCP2(T158M)^{Rho}) for 1 hr at 37 °C, 5% CO_2. Nuclei were identified by incubation with Hoechst 33342 for 5 min at the end of the incubation period. Cells

with nuclear fluorescence are highlighted in the white dash boxes. Scale bar = 10 µm. The results shown are representative of at least two biological replicates.

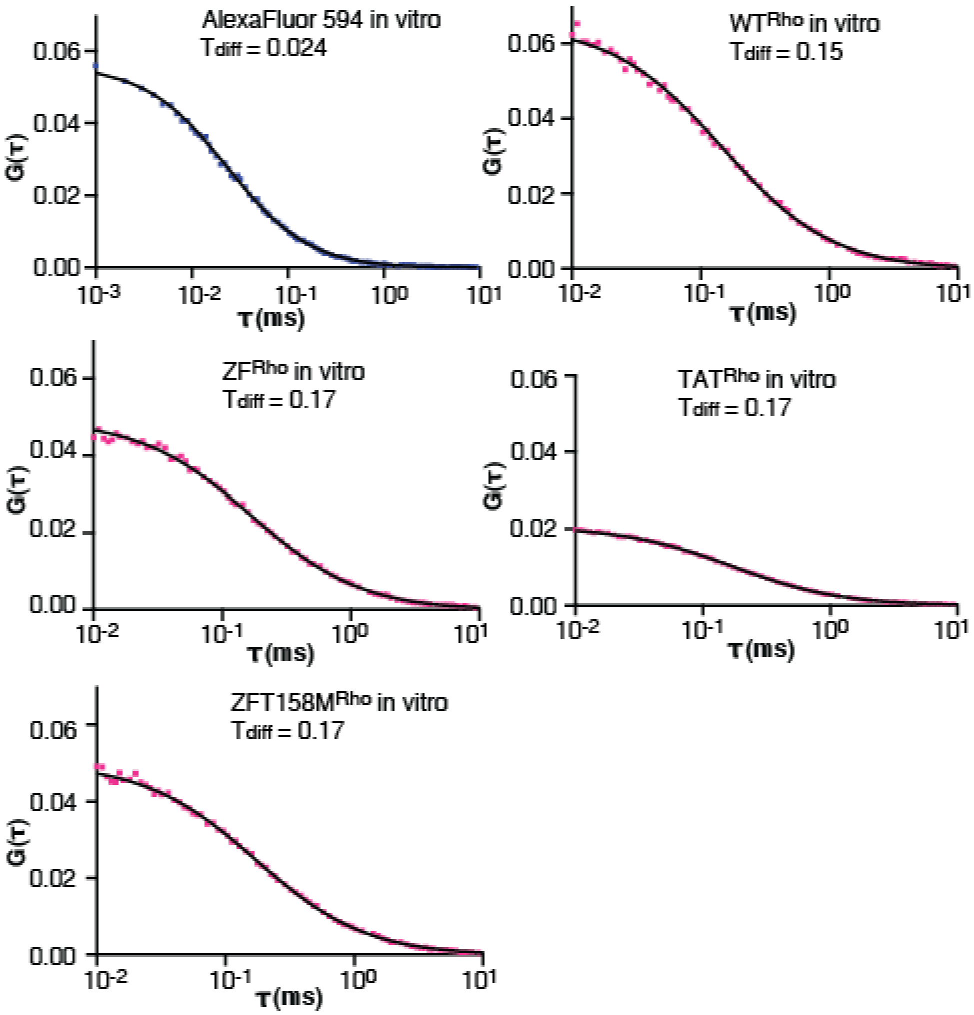

Figure S3. Representative *in vitro* autocorrelation data of 100 nM AlexaFluor594 (in milliQ water, blue) and MeCP2-Rho variants (in DMEM, pink). The diffusion times (τ_{diff}, ms) were obtained by fitting the data to a 3D diffusion model (black curve) based on a previously published protocol.[8]

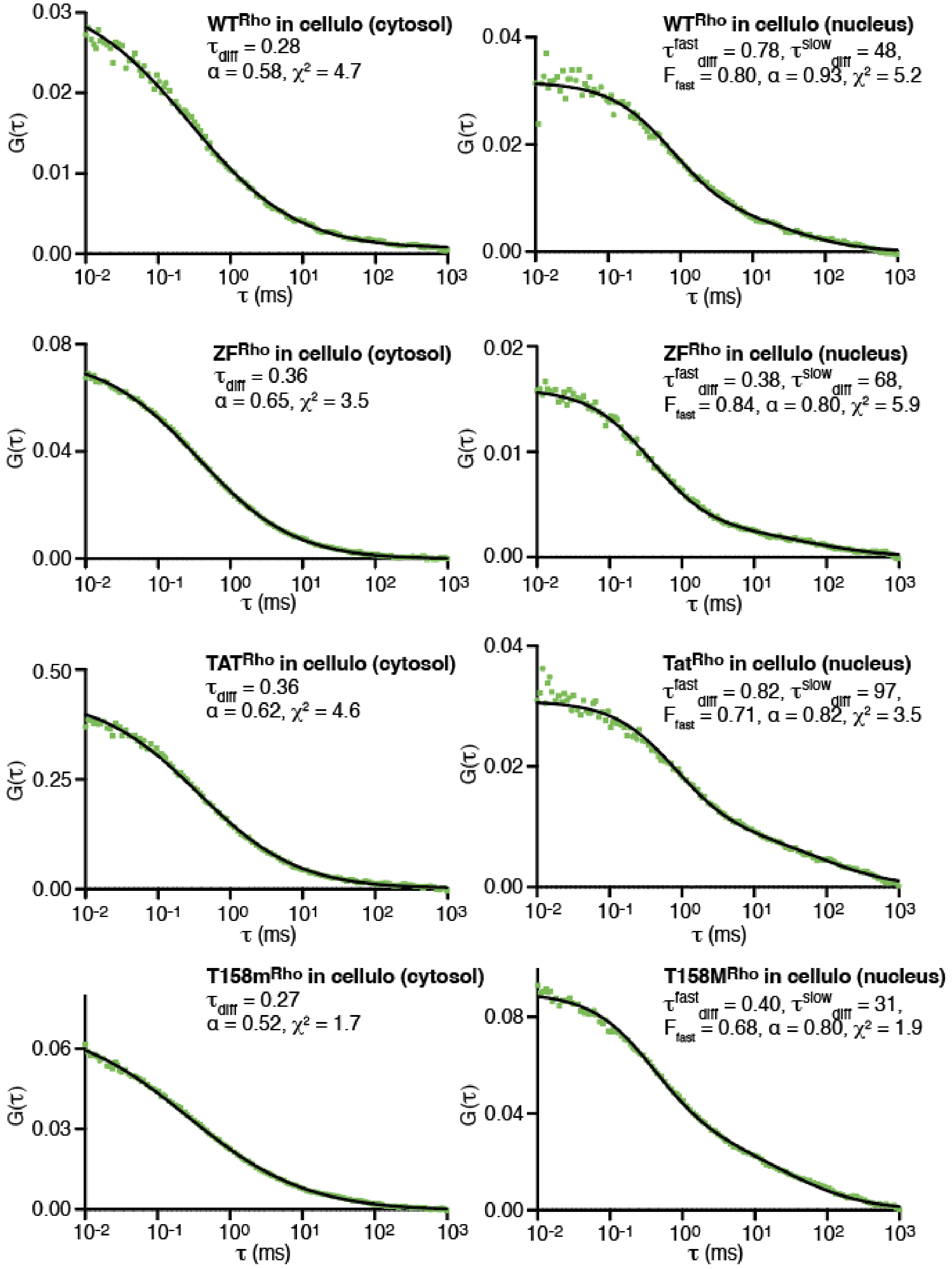

Figure S4. Representative *in cellulo* autocorrelation data of MeCP2-Rho variants in Saos-2 cells. The autocorrelation data (green dots) was fitted to a 3D anomalous diffusion equation[8] (cytosol, black curves) or a two-component 3D diffusion equation (nucleus, black curves)[13] using a custom MATLAB script.

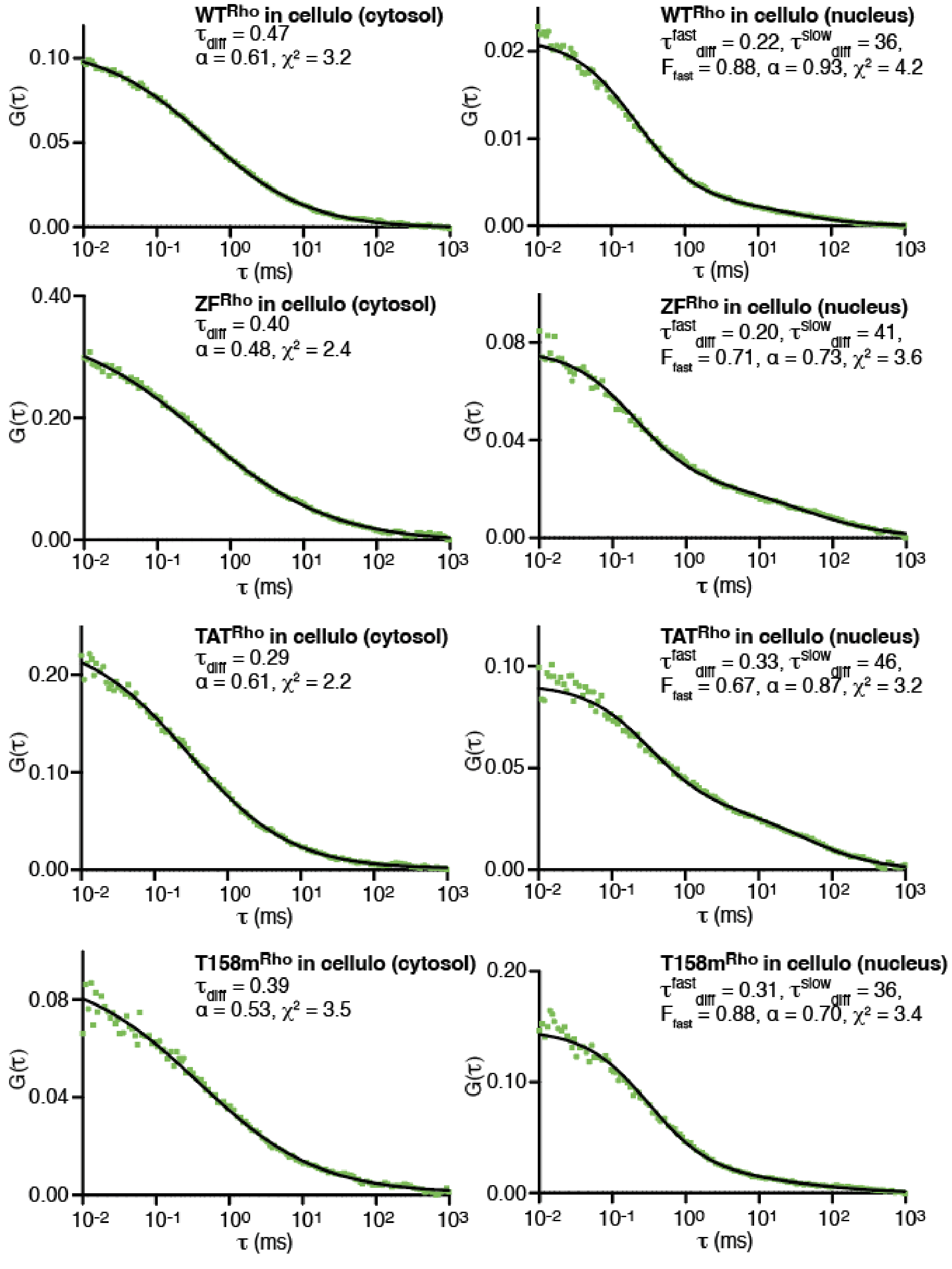

Figure S5. Representative *in cellulo* autocorrelation data of MeCP2-Rho variants in CHO-K1 cells. The autocorrelation data (green dots) was fitted to a 3D anomalous diffusion equation[8] (cytosol, black curves) or a two-component 3D diffusion equation (nucleus, black curves)[13] using a custom MATLAB script.

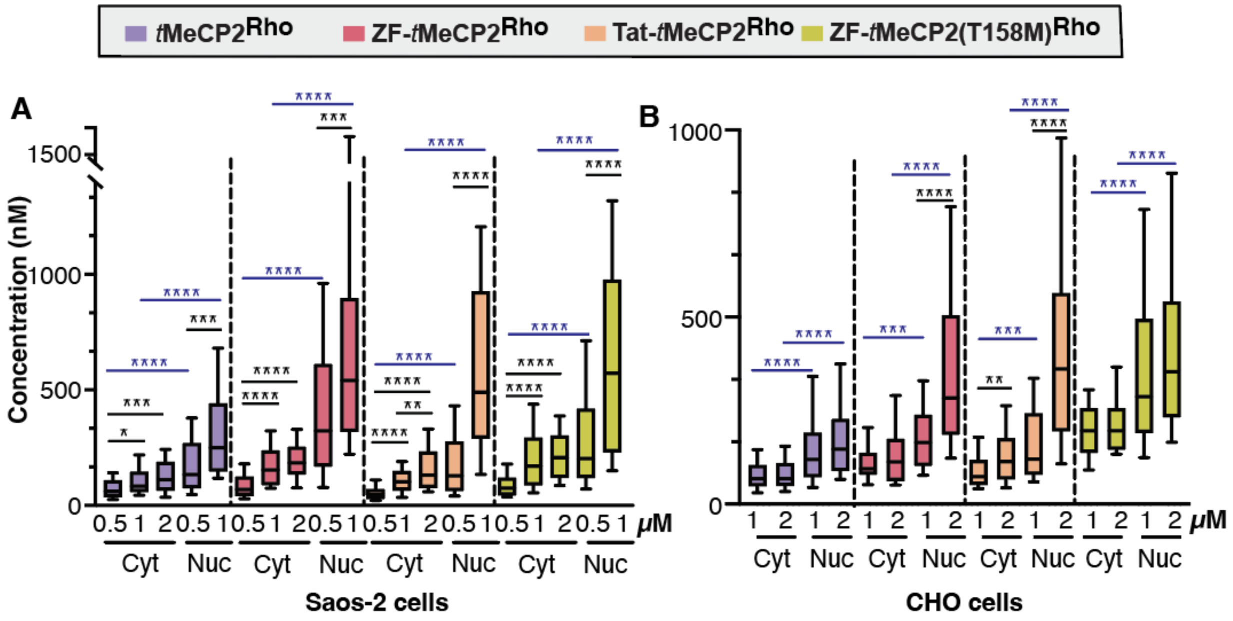

Figure S6. Protein intracellular concentrations determined by FCS. Saos-2 (A) or CHO-K1 (B) cells were incubated with the indicated *t*MeCP2-Rho variants for 1 hr at 37 °C, 5% CO_2. The autocorrelation data (**Figure S4-5**) from FCS measurements was fitted to a 3D anomalous diffusion equation (cytosol)[8] or a two-component 3D diffusion equation (nucleus)[13] to establish the concentration of each protein in the cytosol and nucleus. Center line, median; box limits, 25-75 percentile; whiskers, 10-90 percentile. (n > 30 cells total for each condition from at least two biological replicates). The cytosolic and nuclear concentrations of the same *t*MeCP2-Rho variant under the same treatment condition (e.g. 1 μM) were compared using two tailed unpaired parametric t test with Welch's correction. The dose-dependent concentrations of the same *t*MeCP2-Rho variant under the same location (e.g. WT^{Rho} in the cytosol) were compared using either Brown-Forsythe and Welch ANOVA followed by Dunnett's T3 multiple comparisons test (for cytosolic concentration in Saos-2 cells, P values are adjusted to account for multiple comparisons) or two tailed unpaired parametric t test with Welch's correction (for nuclear concentration in Saos-2 cells and all CHO-K1 cell data) ****$p \leq 0.0001$, ***$p \leq 0.001$,**$p \leq 0.01$, *$p \leq 0.05$.

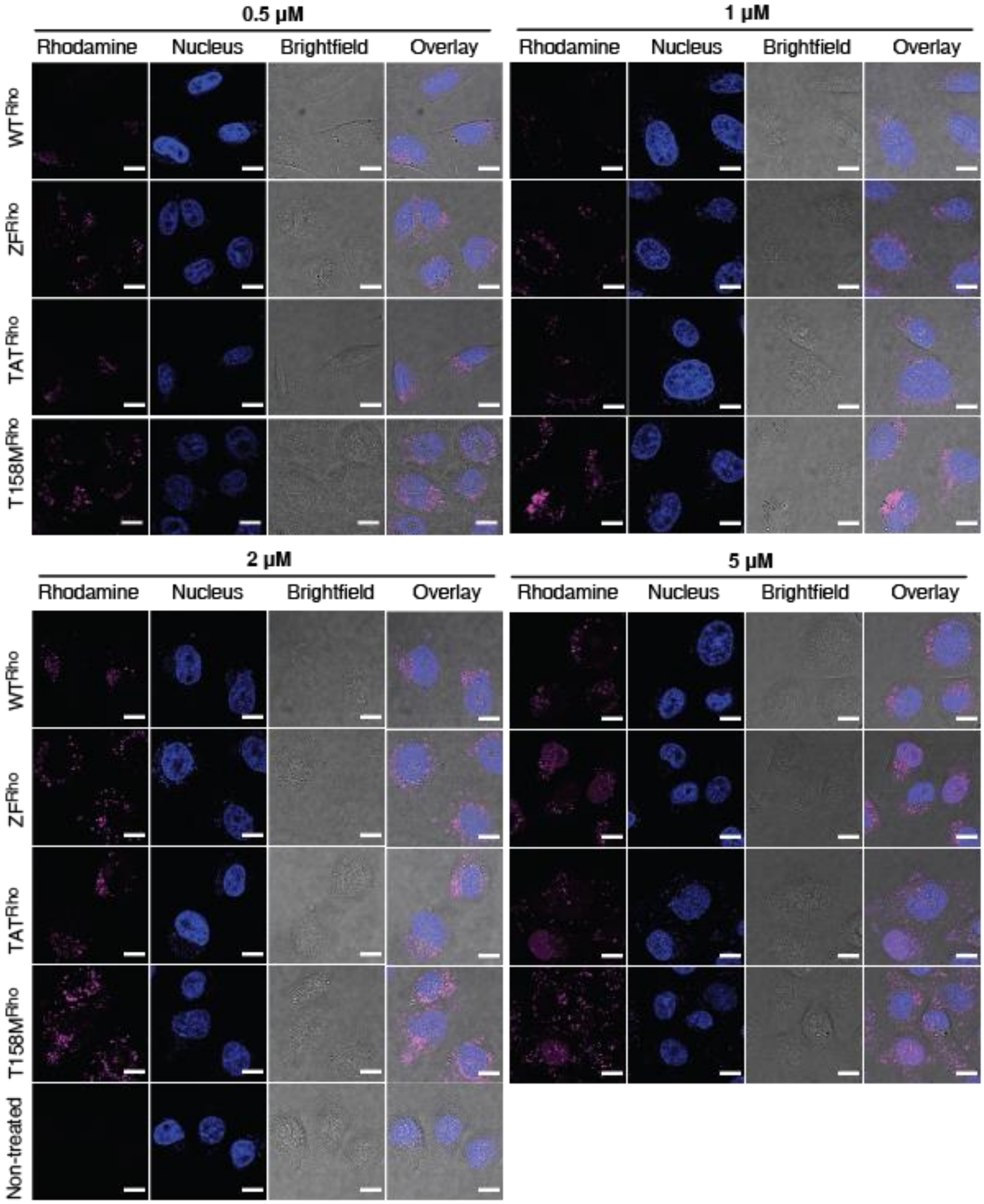

Figure S7. Representative 2D confocal images of CHO-K1 cells treated with *t*MeCP2-Rho variants. Different concentrations of *t*MeCP2-Rho variants (WT^{Rho}: *t*MeCP2^{Rho}, ZF^{Rho}: ZF-*t*MeCP2^{Rho}, Tat^{Rho}: Tat-*t*MeCP2^{Rho}, T158M^{Rho}: ZF-*t*MeCP2(T158M)^{Rho})) were added to the cells and incubated for 1 hr at 37 °C, 5% CO_2. Nuclei were identified by incubation with Hoechst 33342 for 5 min at the end of the incubation period. Scale bar = 10 μm. The results shown are representative of at least two biological repeats.

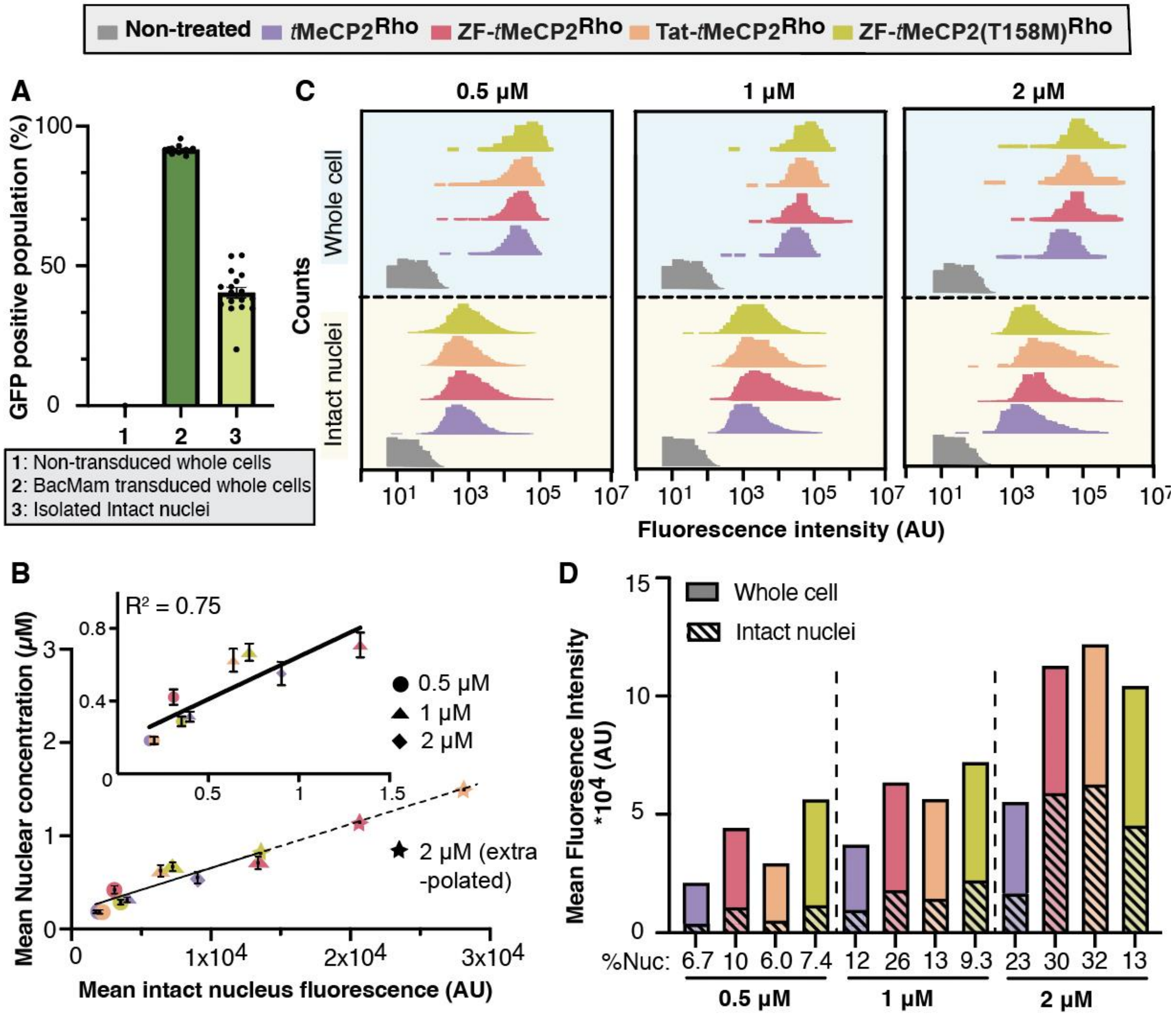

Non-treated
tMeCP2Rho
ZF-tMeCP2Rho
Tat-tMeCP2Rho
ZF-tMeCP2(T158M)Rho
A
GFP positive population (%)
1: Non-transduced whole cells
2: BacMam transduced whole cells
3: Isolated Intact nuclei
C
0.5 μM
1 μM
2 μM
Whole cell
Intact nuclei
Counts
Fluorescence intensity (AU)
B
R² = 0.75
0.5 μM
1 μM
2 μM
2 μM (extra -polated)
Mean Nuclear concentration (μM)
Mean intact nucleus fluorescence (AU)
D
Whole cell
Intact nuclei
Mean Fluoresence Intensity *10^4 (AU)
%Nuc: 6.7 10 6.0 7.4 12 26 13 9.3 23 30 32 13
0.5 μM
1 μM
2 μM

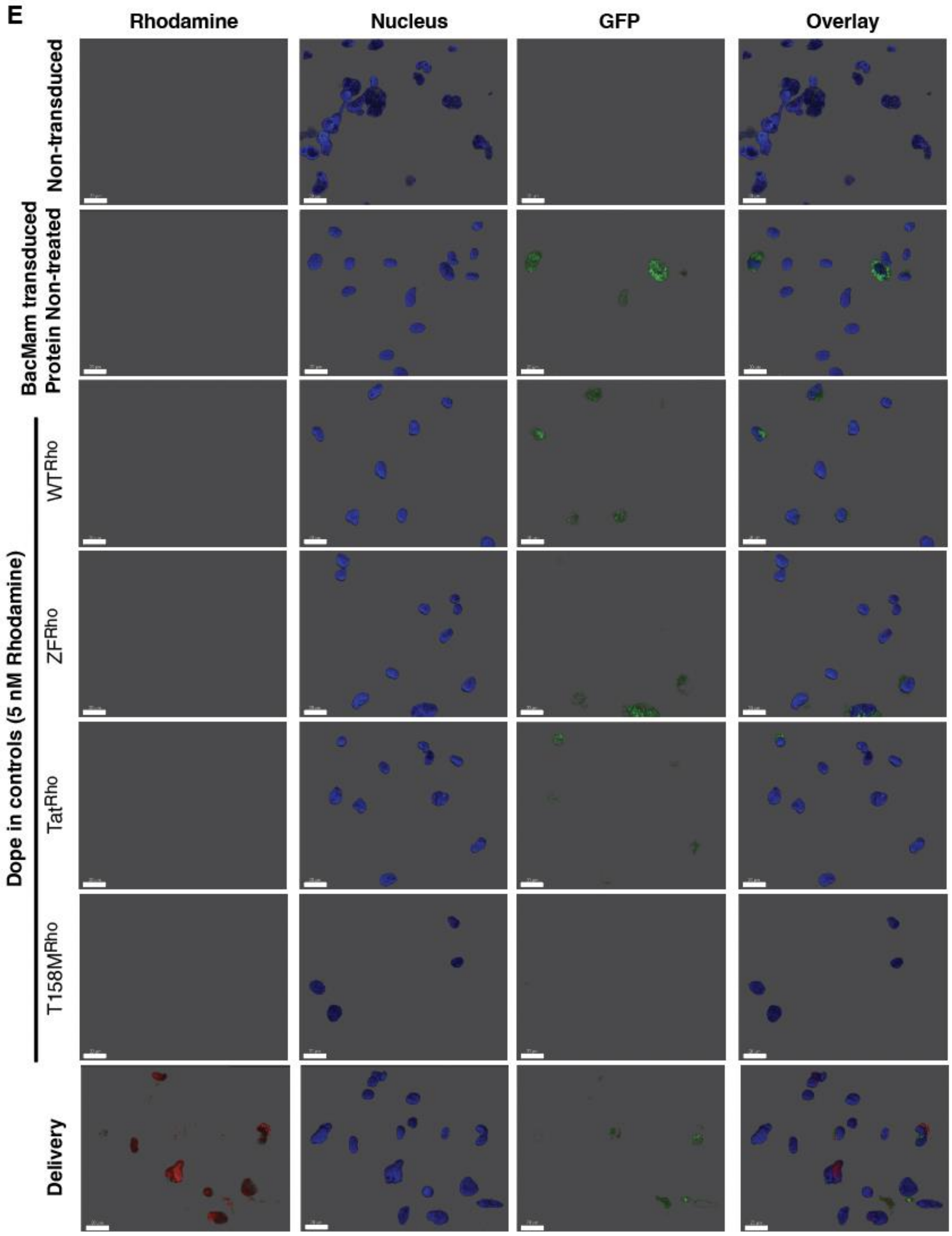

Figure S8. Flow cytometry analysis of Saos-2 cells treated with *t*MeCP2-Rho variants

(A) Percentage of GFP positive population in BacMam transduced whole cell and isolated intact nuclei. (B) Scatter plot depicting the correlation of the mean nuclear fluorescence of intact Saos-2 nuclei measured by flow cytometry (x-axis) and the intra-nuclear concentrations measured by FCS (y-axis). Dots are represented as mean ± SEM. Inset: Data for treatment with 0.5 - 1.0 µM proteins and 2 µM *t*MeCP2-Rho were fitted with a simple linear regression line. The nuclear concentration of ZF-*t*MeCP2-Rho, Tat-*t*MeCP2-Rho and ZF-*t*MeCP2(T158M)Rho in Saos-2 cells treated with 2 µM proteins (stars) were extrapolated based on their corresponding nuclear fluorescence values. (C) Whole cells and intact nucleus rhodamine fluorescence histogram of Saos-2 cells treated with tMeCP2-Rho variants for 1 hr at 37 °C, 5% CO_2 measured by flow cytometry, with the mean rhodamine fluorescence intensity and percentage of nuclear delivery shown in (D). (E) Representative 3D z-stacking images of isolated intact nuclei under different treatment conditions. Scale bar = 20 µm. For dope in controls, *t*MeCP2-Rho variants were mixed with lysis buffer (500 µL) to a final rhodamine concentration of 5 nM (50 fold higher than the theoretical rhodamine protein concentrations in solution after lysis. Calculations were done based on cytosolic concentrations of *t*MeCP2-Rho variants in **Figure S6A**). For the delivery sample (positive control), Saos-2 cells were treated with 1 µM Tat-*t*MeCP2-Rho as described in **Figure 2A** before being lysed and isolated for imaging.

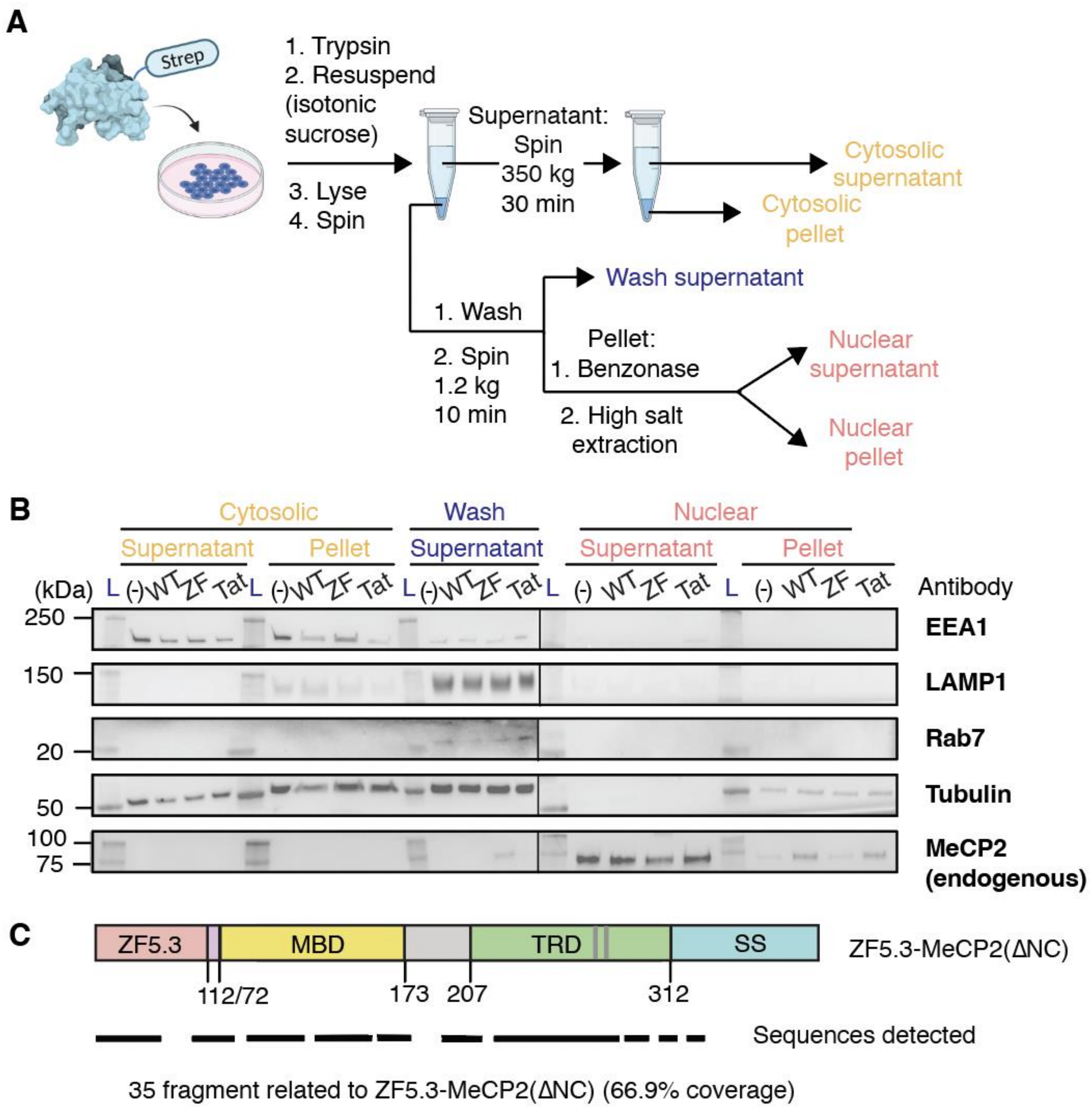

Figure S9. Cellular fractionation and LC-MS/MS analysis of delivered intact proteins. (A) Scheme illustrating the workflow to isolate the cytosolic and nuclear fractions of Saos-2 cells after delivery of 1 µM *t*MeCP2 (WT), ZF-*t*MeCP2 (ZF), Tat-*t*MeCP2 (Tat) for 1 hr at 37 °C, 5% CO_2. One dish of cells with the same clear McCoy's media added was included as a non-treated control (-). Detailed isolation procedures can be found in Methods and Materials. (B) Western blot analysis of different cellular fractions isolated from (A) with antibodies against endocytic (EEA1, LAMP1, Rab7), cytosolic (tubulin) and nuclear (endogenous MeCP2) markers. The gel results shown are representative of three biological repeats. L, Ladder. (C) Scheme illustrating the positions of fragments detected by LC-MS/MS. ZF-*t*MeCP2 isolated from Saos-2 cells as shown in (A) was enriched with MagStrep "type3" XT magnetic beads (IBA 2-4090-002) that recognizes the C terminal Strep-tagII and subjected to trypsin digest before LC-MS/MS analysis.

In total 35 relevant fragments were identified, corresponding to 66.9% of protein coverage. Representative spectra are shown in **Figure S10**.

MYSCNVCGKAPVLSRHLNRHLRVHRRATGSGMVAGMLGLREEKPAVPEASASPKQRRSIIR-
DRGPMYDDPTLPEGWTRKLKQRKSGRSAGKYDVYLINPQGKAFRSKVELIAYFEKVGDTSLDPNDFDFT
VTGRGSPSRREQKPPKKPKSPKAPGTGRGRGRPKGSGTGRPKAAASEGVQVKRVLEKSPGKLVVKMPFQ
ASPGGKGEGGGATTSAQVMVIKRPGRKRKAEADPQAIPKKRGRKPGSVVAAAAAEAKKKAVKESSIRSV
HETVLPIKKRKTRETVGSSGSSGLPETGGWSHPQFEK

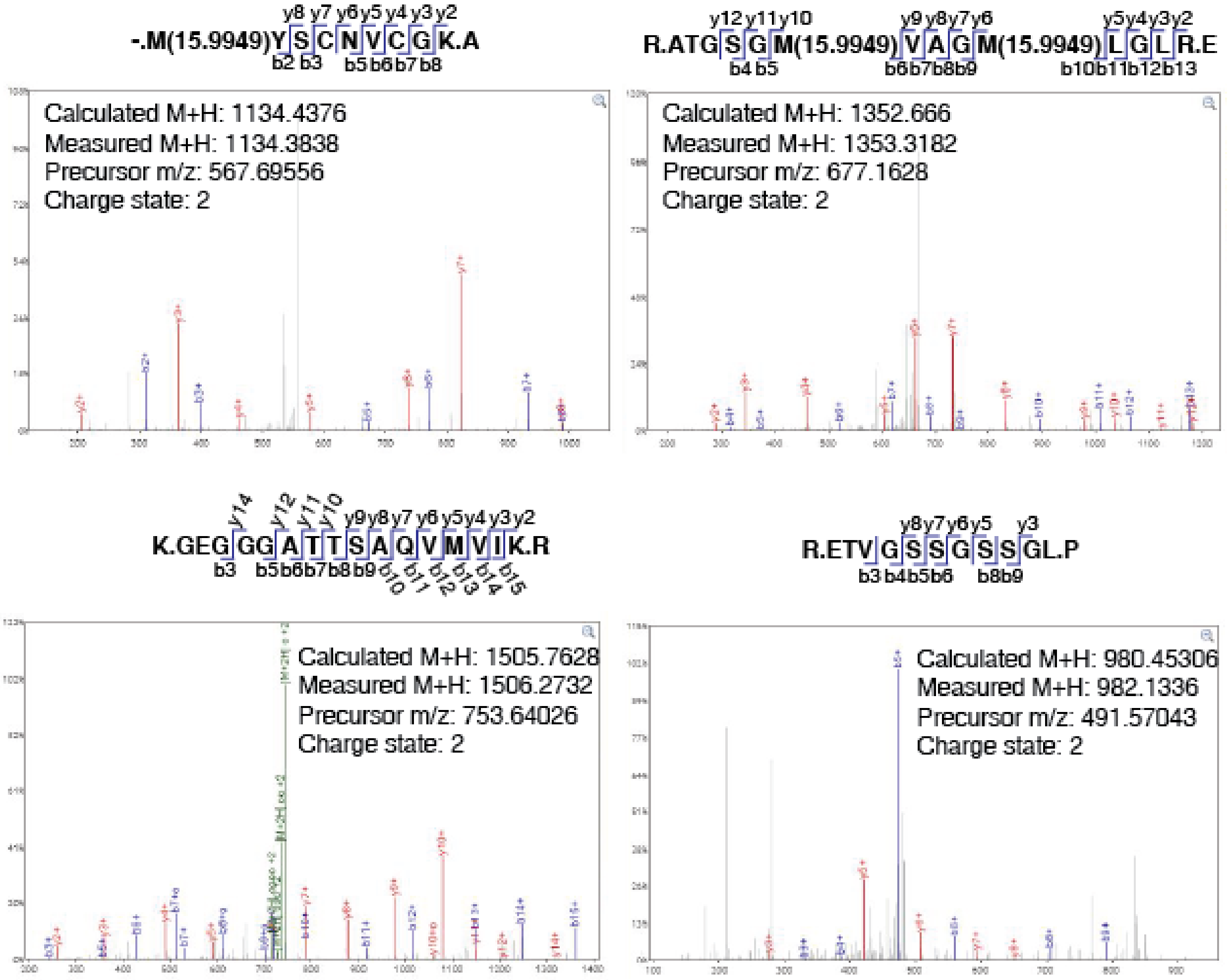

Figure S10. Representative LC-MS/MS spectra of trypsin digested ZF-MeCP2 isolated from Saos-2 cells. The whole sequence of ZF-MeCP2 is shown with fragments detected bolded and selected fragments highlighted in red. The protein was enriched via the underlined Strep-tagII sequence at the C terminus.

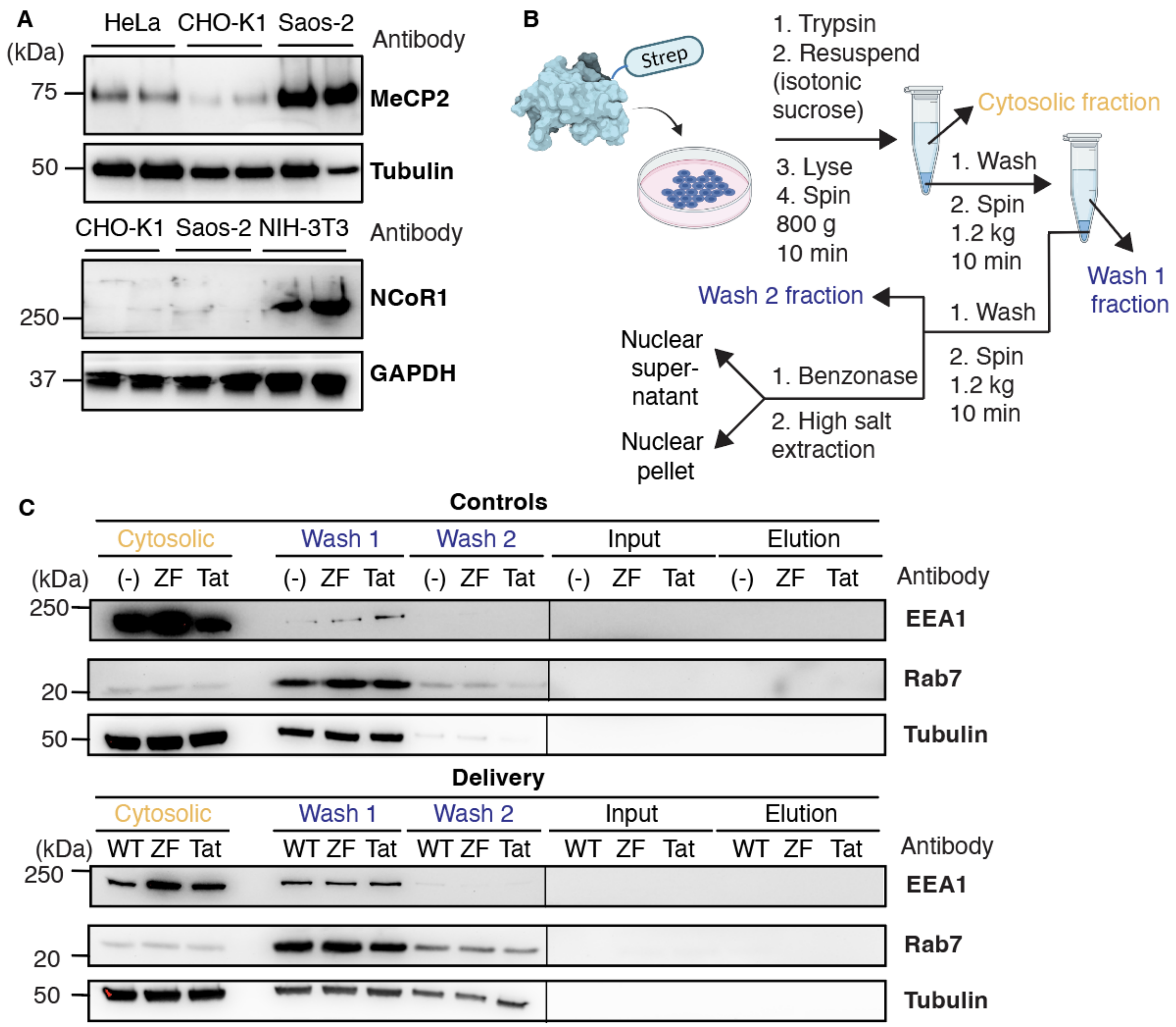

Figure S11. *In cellulo* co-immunoprecipitation assay in CHO-K1 cells

(A) Relative levels of endogenous MeCP2 and NCoR1 in lysates prepared from different cell lines. Two biological replicates were examined per cell line. Western blots were performed using primary antibodies against MeCP2 (3456S), tubulin (CST 2125S), NCoR1 (CST 5948S), GAPDH (2118S) and the secondary antibody HRP-linked Anti-rabbit IgG (CST 7074S). (B) Scheme illustrating the workflow to isolate and extract the nuclear proteins of CHO-K1 cells after incubation with 1 µM *t*MeCP2 (WT), ZF-*t*MeCP2 (ZF), Tat-*t*MeCP2 (Tat) for 1 hr at 37 °C, 5% CO_2. Three dishes of non-treated cells (-) were incubated under identical conditions as controls and later doped with either 150 nM ZF-*t*MeCP2 or Tat-*t*MeCP2 after the nuclear supernatant fractions were isolated. Detailed isolation procedures can be found in Methods and Materials. (C) Western blot analysis of different cellular fractions isolated from (B) with antibodies against endocytic (EEA1, Rab7) and cytosolic (tubulin) markers. The gel results shown are representative of two biological repeats.

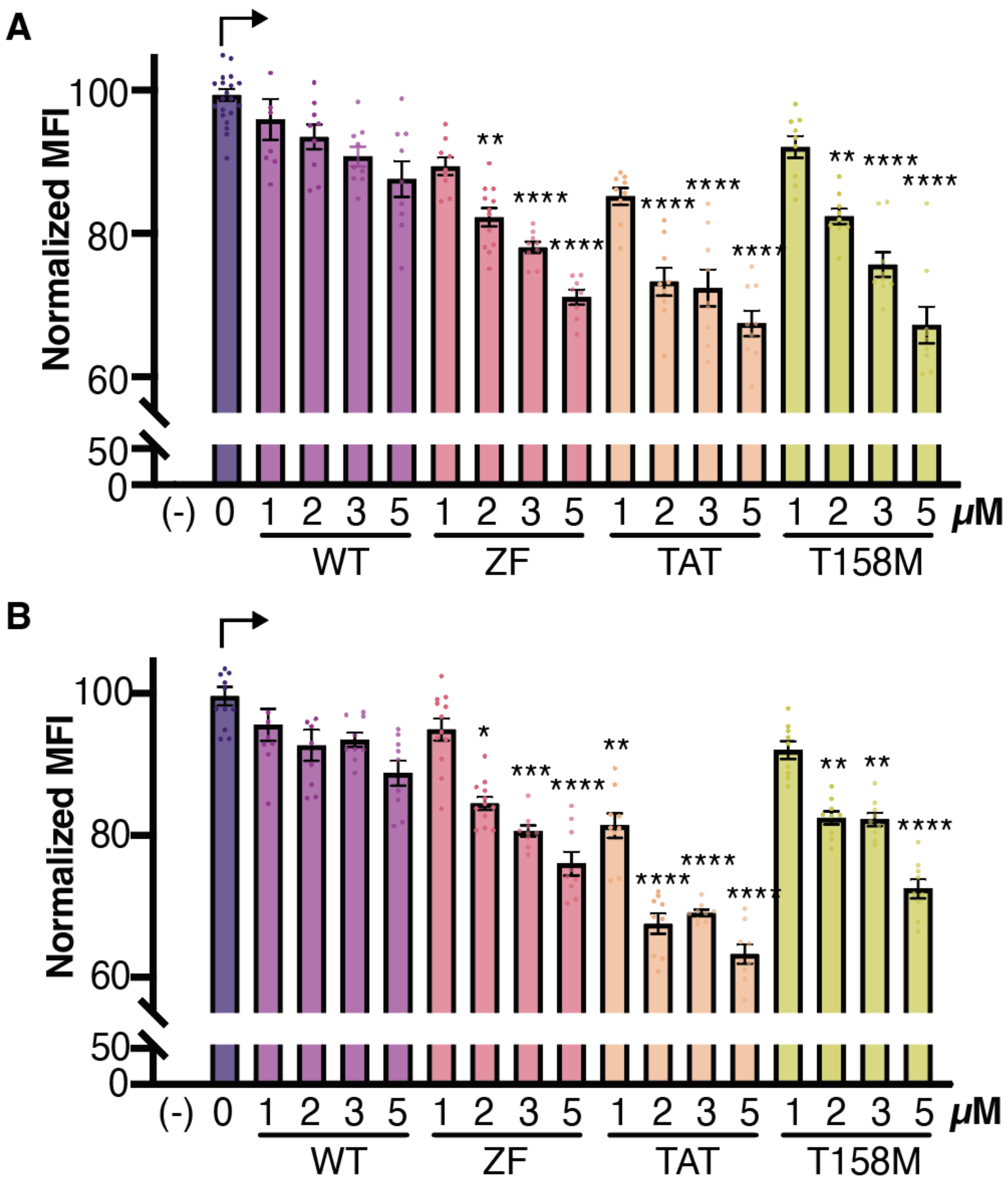

Figure S12. Mean fluorescence intensities (MFI) of CHO-K1 cells studied in transcription repression assay. CHO-K1 cells transfected with methylated (A) or non-methylated (B) reporter plasmids were incubated with 1-5 μM the indicated tMeCP2 variants (WT: tMeCP2, ZF: ZF-tMeCP2, Tat: Tat-tMeCP2, P: ZF-tMeCP2(P302L)) for 1 hr at 37 °C, 5% CO_2 and analyzed using flow cytometry to identify changes in green fluorescence levels. Cells were gated for green fluorescence based on the background fluorescence level of cells that were not transfected with a reporter plasmid. Data are represented as mean ± SEM. Each sample comprised 130 μL (at least 50,000 cells) and at least three technical and biological replicates at each condition. The MFI

was normalized against non-treated cells transfected with the reporter plasmid. Statistical significance compared with non-treated cells transfected with the reporter plasmid was calculated using a nonparametric Kruskal-Wallis test followed by Dunnett's T3 multiple comparisons test. ****$p \leq 0.0001$, ***$p \leq 0.001$,**$p \leq 0.01$, *$p \leq 0.05$. Each P value is adjusted to account for multiple comparisons.

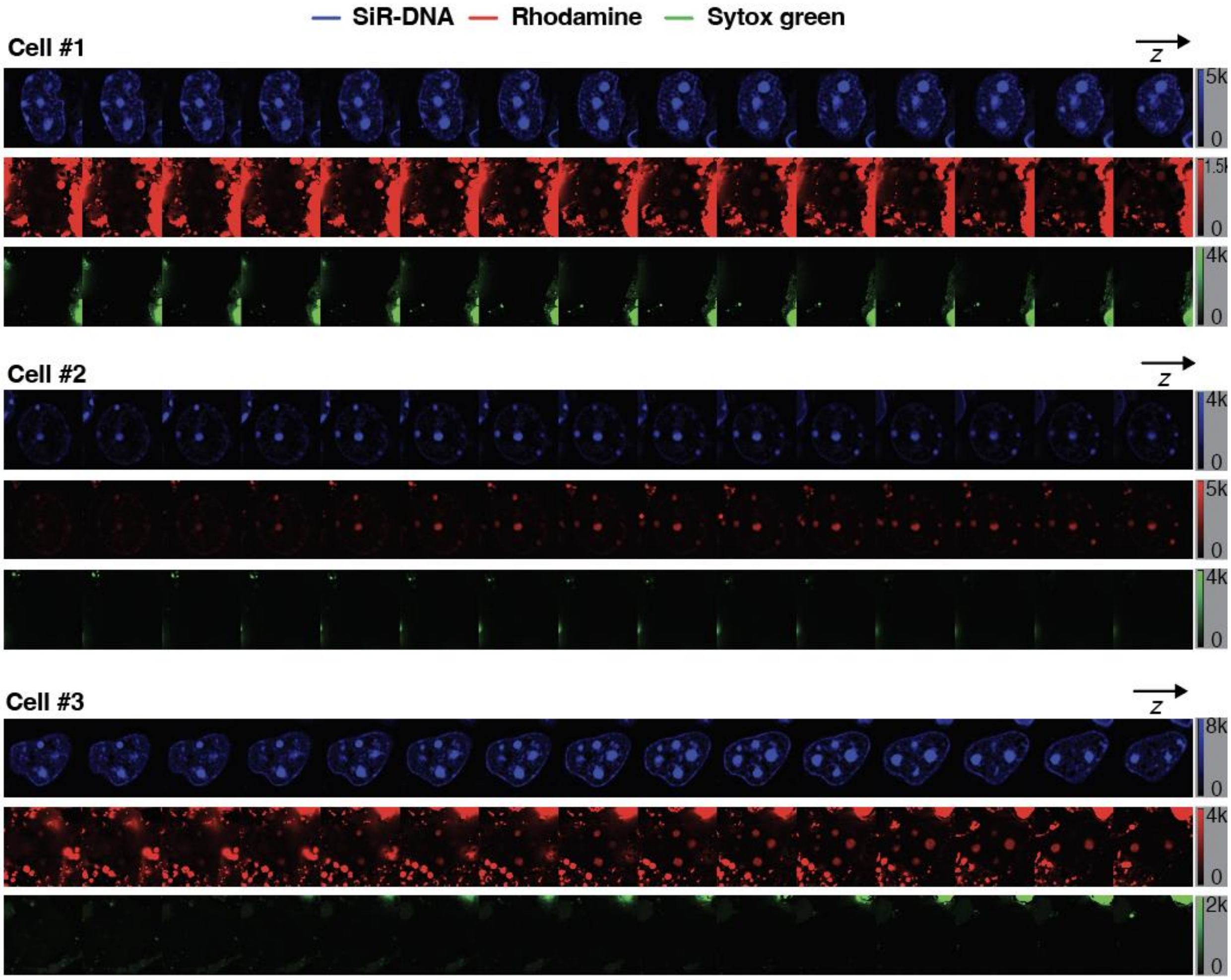

Figure S13. Representative super-resolution z-stack images of ZF-*t*MeCP2 delivered mouse primary cortical neurons. Neurons were sequentially incubated with SiR-DNA live cell nuclear stain (1 h, 37 °C, 5% CO_2), 1 µM ZF-*t*MeCP2-Rho protein (1 h, 37 °C, 5% CO_2) and SYTOX® Green dye (10 min, 37 °C, 5% CO_2) before imaged on a Zeiss LSM 900 laser-scanning microscope. Brightness and contrast of each channel was adjusted for visualization purposes, with the upper and lower thresholds indicated on each image.

	**Helix**	**Anti-parallel**	**Parallel**	**Turn**	**Others**
***t*MeCP2**	2.3%	20.8%	0%	19.5%	57.5%
**ZF-*t*MeCP2**	2.0%	20.4%	0%	19.8%	57.8%
**Tat-*t*MeCP2**	1.9%	22.1%	0%	19.2%	56.8%
**ZF-*t*MeCP2 (T158M)**	2.2%	21.4%	0%	19.5%	57%
**ZF-*t*MeCP2 (P302L)**	1.6%	22.3%	0%	21%	55.2%

Table S1. Secondary structure predictions of *t*MeCP2 variants. Predictions were generated using BeStSel (http://bestsel.elte.hu/index.php) based on the circular dichroism spectra of proteins in **Figure S1C**.

		*t*MeCP2	ZF-*t*MeCP2	Tat-*t*MeCP2	ZF-*t*MeCP2 (T158M)
**In vitro measurements**	T_{diff} (ms)	0.154 ± 0.003	0.171 ± 0.001	0.176 ± 0.003	0.175 ± 0.001
		**Saos-2 cells**			
		***t*MeCP2**	**ZF-*t*MeCP2**	**Tat-*t*MeCP2**	**ZF-*t*MeCP2 (T158M)**
**0.5 µM treatment in cytosol**	T_{diff} (ms)	0.246 ± 0.015	0.273 ± 0.025	0.299 ± 0.020	0.313 ± 0.024
**1 µM treatment in cytosol**	T_{diff} (ms)	0.342 ± 0.020	0.363 ± 0.028	0.378 ± 0.025	0.338 ± 0.020
**2 µM treatment in cytosol**	T_{diff} (ms)	0.278 ± 0.020	0.402 ± 0.050	0.365 ± 0.022	0.338 ± 0.022
**0.5 µM treatment in nucleus**	$T_{diff\text{-}fast}$ (ms)	0.300 ± 0.024	0.280 ± 0.019	0.380 ± 0.023	0.286 ± 0.019
	$T_{diff\text{-}slow}$ (ms)	50.8 ± 13.0	38.5 ± 8.75	40.9 ± 8.31	29.4 ± 6.88
	F_{fast}	0.775 ± 0.023	0.805 ± 0.018	0.778 ± 0.023	0.763 ± 0.021
	F_{slow}	0.225 ± 0.023	0.195 ± 0.018	0.222 ± 0.023	0.237 ± 0.021
	α: α<1	0.759 ± 0.031	0.786 ± 0.030	0.796 ± 0.028	0.769 ± 0.026
	α: % α<1	66.7	74.2	78.8	82.5
**1 µM treatment in nucleus**	$T_{diff\text{-}fast}$ (ms)	0.426 ± 0.028	0.398 ± 0.024	0.494 ± 0.019	0.373 ± 0.016
	$T_{diff\text{-}slow}$ (ms)	59.1 ± 8.51	97.1 ± 11.5 (vs T158M: ****)	83.5 ± 8.45 (vs T158M: ****)	38.3 ± 4.18
	F_{fast}	0.745 ± 0.021	0.692 ± 0.022	0.682 ± 0.013	0.631 ± 0.020 (vs WT: ***)
	F_{slow}	0.255 ± 0.021	0.308 ± 0.022	0.318 ± 0.013	0.369 ± 0.020 (vs WT: ***)
	α: α<1	0.728 ± 0.0027	0.780 ± 0.025	0.846 ± 0.023	0.806 ± 0.020
	α: % α<1	69.2	64.7	50.9	62.8
**2 µM treatment in nucleus**	$T_{diff\text{-}fast}$ (ms)	0.411 ± 0.028	N/A	N/A	N/A
	$T_{diff\text{-}slow}$ (ms)	50.7 ± 8.32			
	F_{fast}	0.674 ± 0.026			
	F_{slow}	0.326 ± 0.026			
	α: α<1	0.765 ± 0.027			
	α: % α<1	57.1			

Table S2. Fitting parameters for FCS measurements *in vitro* and in Saos-2 cells. The autocorrelation curves obtained from the *in vitro* measurements (**Figure S3**) were fitted to a 3D diffusion equation and the *in vivo* measurements (**Figure S4**) were fitted to a 3D anomalous diffusion equation (cytosol) and a two-component 3D diffusion equation (nucleus). Values are represented in mean ± SEM. (n>20 from at least two biological replicates). The values of $T_{diff\text{-}slow}$, F_{fast} and F_{slow} in each condition are compared using Brown-Forsythe and Welch ANOVA followed by Dunnett's T3 multiple comparisons test. ****$p \leq 0.0001$, ***$p \leq 0.001$,**$p \leq 0.01$, *$p \leq 0.05$.

CHO cells						
			***t*MeCP2**	**ZF-*t*MeCP2**	**Tat-*t*MeCP2**	**ZF-*t*MeCP2 (T158M)**
**1 µM treatment in cytosol**	τ_{diff} (ms)		0.296 ± 0.024	0.393 ± 0.043	0.407 ± 0.051	0.369 ± 0.048
**2 µM treatment in cytosol**	τ_{diff} (ms)		0.445 ± 0.046	0.361 ± 0.035	0.388 ± 0.042	0.332 ± 0.033
**1 µM treatment in nucleus**	$\tau_{diff\text{-}fast}$ (ms)		0.386 ± 0.021	0.397 ± 0.026	0.407 ± 0.032	0.387 ± 0.023
	$\tau_{diff\text{-}slow}$ (ms)		68.5 ± 11.8	56.5 ± 7.32	55.4 ± 10.1	62.8 ± 6.59
	F_{fast}		0.839 ± 0.010<br>(vs ZF: **)	0.737 ± 0.026	0.776 ± 0.027	0.837 ± 0.010<br>(vs ZF: **)
	F_{slow}		0.161 ± 0.010<br>(vs ZF: **)	0.263 ± 0.026	0.224 ± 0.027	0.163 ± 0.010<br>(vs ZF: **)
	α	α<1	0.786 ± 0.027	0.797 ± 0.027	0.778 ± 0.030	0.799 ± 0.025
		% α<1	49	48.8	50	42.1
**2 µM treatment in nucleus**	$\tau_{diff\text{-}fast}$ (ms)		0.343 ± 0.019	0.365 ± 0.022	0.354 ± 0.019	0.432 ± 0.017
	$\tau_{diff\text{-}slow}$ (ms)		54.6 ± 6.9	54.5 ± 5.78	54 ± 6.26	61.2 ± 3.89
	F_{fast}		0.818 ± 0.012	0.751 ± 0.021<br>(vs WT: *)<br>(vs TAT: *)	0.663 ± 0.020<br>(vs WT: ****)	0.744 ± 0.017<br>(vs WT: **)<br>(vs TAT: *)
	F_{slow}		0.182 ± 0.012	0.249 ± 0.021<br>(vs WT: *)<br>(vs TAT: *)	0.337 ± 0.020<br>(vs WT: ****)	0.256 ± 0.017<br>(vs WT: **)<br>(vs TAT: *)
	α	α<1	0.772± 0.025	0.788 ± 0.024	0.792 ± 0.025	0.833 ± 0.019
		% α<1	53.6	60.9	54.7	41.2

Table S3. Fitting parameters for FCS measurements and in CHO cells. The autocorrelation curves obtained from the *in vivo* measurements (**Figure S5**) were fitted to a 3D anomalous diffusion equation (cytosol) and a two-component 3D diffusion equation (nucleus). Values are represented in mean ± SEM. (n>20 from at least two biological replicates). The values of $T_{diff\text{-}slow}$, F_{fast} and F_{slow} in each condition are compared using Brown-Forsythe and Welch ANOVA followed by Dunnett's T3 multiple comparisons test. ****$p \leq 0.0001$, ***$p \leq 0.001$,**$p \leq 0.01$, *$p \leq 0.05$.

Fitting equation	*t*MeCP2	ZF-*t*MeCP2	Tat-*t*MeCP2	ZF-*t*MeCP2 (T158M)
Eq 3	7.3 ± 1.6	5.2 ± 1.1	7.1 ± 0.87	7.5 ± 0.88
Eq 4	13 ± 4.6	7.3 ± 1.6	8.3 ± 0.9	11 ±1.3
Eq 2	13 ± 4.4	9.2 ± 2.2	9.4 ± 2	12 ±2.6

Table S4. Fitting chi-squares for different equations
The autocorrelation curves obtained *in vivo* measurements in the nucleus was fitted with 1) a two component 3D diffusion equation with one free and one anomalous component (equation 3 in the methods section); 2) a two component 3D diffusion equation with two free components (equation 4 in the methods section); or 3) a one component 3D anomalous equation (equation 2 in the methods section). Chi-square for each fitting was calculated. Values are represented in mean ± SEM.

Table S5. Relevant primers and sequences

**Cloning to generate tMeCP2 variants: DNA sequence**	
ZF5.3-*t*MeCP2(ΔNC)-LPETGG-Strep protein	TGAGCGGATAACAATTCCCCTCTAGAAATAATTTTGTTTAACT TTAAGAAGGAGATATACCATGTACAGCTGCAACGTGTGTGGG AAAGCTTTTGTACTTTCGCGCCACTTGAACCGCCACTTACGTG TACATCGTCGCGCGACTGGTAGCGGGATGGTAGCTGGCATGC TGGGTCTTCGCGAAGAGAAACCAGCCGTTCCGGAAGCATCGG CGTCGCCCAAGCAACGTCGCAGCATCATCCGCGACCGCGGCC CCATGTACGATGATCCGACTTTACCTGAAGGTTGGACACGTA AACTGAAGCAACGTAAGAGCGGGCGTTCAGCGGGGAAGTAT GATGTTTATTTGATTAACCCACAGGGGAAGGCTTTCCGCTCTA AGGTTGAGCTTATCGCCTACTTCGAGAAAGTGGGAGATACCT CGCTGGATCCGAACGATTTCGATTTTACAGTCACAGGCCGTGG CTCCCCATCCCGCCGCGAGCAGAAGCCTCCGAAAAAACCAAA ATCTCCTAAGGCGCCAGGCACTGGACGTGGTCGCGGGCGTCC TAAGGGCAGCGGTACCGGACGCCCAAAAGCAGCTGCCTCCGA AGGAGTACAAGTGAAACGTGTTTTAGAAAAGAGTCCAGGAAA ACTTGTTGTGAAAATGCCCTTTCAGGCGAGCCCGGGGGGCAA AGGCGAGGGCGGCGGCGCCACAACATCTGCTCAGGTAATGGT TATCAAACGTCCTGGCCGTAAGCGTAAAGCCGAAGCGGACCC TCAAGCAATTCCAAAGAAACGCGGTCGTAAACCAGGCTCGGT AGTAGCCGCTGCGGCGGCTGAAGCTAAAAAGAAGGCCGTTAA

	GGAGTCTAGCATTCGCAGTGTGCACGAGACAGTTTTACCGATCAAGAAACGTAAGACTCGTGAGACCGTTGGCTCTAGCGGGTCCTCAGGTCTTCCGGAAACTGGAGGGTGGAGTCACCCACAATTCGAGAAATAACGGATCCGAATTCGAGCGCCGTCGACAAGCT
Generating *t*MeCP2(ΔNC)-LPETGG-Strep from ZF5.3-tMeCP2(ΔNC)-LPETGG-Strep	TGAGCGGATAACAATTCCCCTCTAGAAATAATTTTGTTTAACTTTAAGAAGGAGATATACCATGGTAGCTGGCATGCTGGGTCTTCGCGAAGAGAAACCAGCCGTTCCGGAAGCATCGGCGTCGCCCAAGCAACGTCGCAGCATCATCCGCGACCGCGGCCCCATGTACGATGATCCGA
Generating Tat-*t*MeCP2(ΔNC)-LPETGG-Strep from ZF5.3-tMeCP2(ΔNC)-LPETGG-Strep	TGAGCGGATAACAATTCCCCTCTAGAAATAATTTTGTTTAACTTTAAGAAGGAGATATACCATGTACGGTCGCAAAAAACGCCGCCAACGTCGTCGCGGTTCCGGTATGGTAGCTGGCATGCTGGGTCTTCGCGAAGAGAAACCAGCCGTTCCGGAAGCATCGGCGTCGCCCAAGCAACGTCGCAGCATCATCCGCGACCGCGGCCCCATGTACGATGATCCGA
Primers for Q5 Site-Directed Mutagenesis to generate ZF5.3-*t*MeCP2(ΔNC)-T158M-LPETGG-Strep	F: TTTCGATTTTatgGTCACAGGCCGTGGC<br>R: TCGTTCGGATCCAGCGAG
Primers for Q5 Site-Directed Mutagenesis to generate ZF5.3-tMeCP2(ΔNC)-P302L-LPETGG-Strep	F: GACAGTTTTActgATCAAGAAACGTAAG<br>R: TCGTGCACACTGCGAATG
**Protein sequences of *t*MeCP2 variants**	
*t*MeCP2(ΔNC)-LPETGG-Strep	MVAGMLGLREEKPAVPEASASPKQRRSIIRDRGPMYDDPTLPEGWTRKLKQRKSGRSAGKYDVYLINPQGKAFRSKVELIAYFEKVGDTSLDPNDFDFTVTGRGSPSRREQKPPKKPKSPKAPGTGRGRGRPKGSGTGRPKAAASEGVQVKRVLEKSPGKLVVKMPFQASPGGKG

	EGGGATTSAQVMVIKRPGRKRKAEADPQAIPKKRGRKPGSVVA AAAAEAKKKAVKESSIRSVHETVLPIKKRKTRETVGSSGSSGLPE TGGWSHPQFEK*
ZF5.3-*t*MeCP2(ΔNC)-LPETGG-Strep	MYSCNVCGKAFVLSRHLNRHLRVHRRATGSGMVAGMLGLREE KPAVPEASASPKQRRSIIRDRGPMYDDPTLPEGWTRKLKQRKSG RSAGKYDVYLINPQGKAFRSKVELIAYFEKVGDTSLDPNDFDFT VTGRGSPSRREQKPPKKPKSPKAPGTGRGRGRPKGSGTGRPKAA ASEGVQVKRVLEKSPGKLVVKMPFQASPGGKGEGGGATTSAQV MVIKRPGRKRKAEADPQAIPKKRGRKPGSVVAAAAAEAKKKAV KESSIRSVHETVLPIKKRKTRETVGSSGSSGLPETGGWSHPQFEK*
Tat-*t*MeCP2(ΔNC)-LPETGG-Strep	MYGRKKRRQRRRGSGMVAGMLGLREEKPAVPEASASPKQRRSI IRDRGPMYDDPTLPEGWTRKLKQRKSGRSAGKYDVYLINPQGK AFRSKVELIAYFEKVGDTSLDPNDFDFTVTGRGSPSRREQKPPKK PKSPKAPGTGRGRGRPKGSGTGRPKAAASEGVQVKRVLEKSPG KLVVKMPFQASPGGKGEGGGATTSAQVMVIKRPGRKRKAEADP QAIPKKRGRKPGSVVAAAAAEAKKKAVKESSIRSVHETVLPIKK RKTRETVGSSGSSGLPETGGWSHPQFEK*
ZF5.3-*t*MeCP2(ΔNC)-T158M-LPETGG-Strep	MYSCNVCGKAFVLSRHLNRHLRVHRRATGSGMVAGMLGLREE KPAVPEASASPKQRRSIIRDRGPMYDDPTLPEGWTRKLKQRKSG RSAGKYDVYLINPQGKAFRSKVELIAYFEKVGDTSLDPNDFDFM VTGRGSPSRREQKPPKKPKSPKAPGTGRGRGRPKGSGTGRPKAA ASEGVQVKRVLEKSPGKLVVKMPFQASPGGKGEGGGATTSAQV MVIKRPGRKRKAEADPQAIPKKRGRKPGSVVAAAAAEAKKKAV KESSIRSVHETVLPIKKRKTRETVGSSGSSGLPETGGWSHPQFEK*
ZF5.3-*t*MeCP2(ΔNC)-P302L-LPETGG-Strep	MYSCNVCGKAFVLSRHLNRHLRVHRRATGSGMVAGMLGLREE KPAVPEASASPKQRRSIIRDRGPMYDDPTLPEGWTRKLKQRKSG RSAGKYDVYLINPQGKAFRSKVELIAYFEKVGDTSLDPNDFDFT VTGRGSPSRREQKPPKKPKSPKAPGTGRGRGRPKGSGTGRPKAA ASEGVQVKRVLEKSPGKLVVKMPFQASPGGKGEGGGATTSAQV MVIKRPGRKRKAEADPQAIPKKRGRKPGSVVAAAAAEAKKKAV KESSIRSVHETVLLIKKRKTRETVGSSGSSGLPETGGWSHPQFEK*
**DNA probes used for fluorescence polarization**	
Methylated	Strand 1: 5’/56-FAM/CCCTATAA/Me-dC/GGAATTCATAATG-3’<br>Strand 2: 5’-CATTATGAATTC/Me-dC/GTTATAGGG-3’

Non-methylated	Strand 1: 5’/56-FAM/CCCTATAACGGAATTCATAATG-3’<br>Strand 2: 5’-CATTATGAATTCCGTTATAGGG-3’
**siRNA used in RNAi**	
RISC-free knockdown	siGENOME RISC-Free Control siRNA: D-001220-01 (Dharmacon)
KIF11 knockdown	siGENOME human SMARTpool siRNA KIF11: D-001220-01 (Dharmacon)
VPS39 knockdown	siGENOME human SMARTpool siRNA VPS39: M-014052-01 (Dharmacon)
TGFBRAP1 knockdown	siGENOME human SMARTpool siRNA TGFBRAP1: M-006903-01 (Dharmacon)
**Gene-specific primers for RT-qPCR**	
GAPDH amplification	PrimeTime qPCR primers Hs.PT.39a.22214836 (IDT)
TGFBRAP1 amplification	Forward: CTGACCACTCAGTACATCATCC<br>Reverse: CTCCTGTCTCCCTATCCTCTT
VPS39 amplification	Forward: AGGGTCTGGCTATTCCTTATCT<br>Reverse: CCTTGCACCTTCTCACAGTATAG
**Cloning to generate reporter plasmids for *in cellulo* transcription assay**	
Primers to amplify mNeonGreen gene	Forward:<br>AGCGAGGAAGGTCAGAAGCTTGGCAATCCGGTACTGGCCACCATGGTGAGCAAGGGCGA<br>Reverse:<br>GGCGGAAAGCCGTGAGAATTCGCCTTGTACAGCTCGTCCATGC
Primers to amplify SNRPN promoter	Forward:<br>CCGGTACCTGAGCTCGCTAGCTTTCTGTACAGAATTTTAAGTATCTGACAATTT<br>Reverse:<br>CAGTACCGGATTGCCAAGCTTCTGACCTTCCTCGCTCCATTG

Supplementary Methods 1. Protein expression and purification

The plasmids encoding *t*MeCP2 variants were each transformed into *E. coli* BL21-CodonPlus(DE3)-RP cells (Agilent, #230255) and selected on a kanamycin (Kan)-chloramphenicol (Cm)-double resistant LB agar plate. For protein expression, a starter culture was generated by inoculating a colony in 25 mL of LB media with 25µL each 1000X Kan and Cm stock solutions. After overnight incubation at 37 °C with shaking at 200 rpm, 20 mL starter culture was transferred into 2 L of LB with Kan and Cm. The culture was grown at 37 °C until OD_{600} = 0.6. At this point, 1 mM IPTG was added to induce expression, and growth continued at 30°C for 3h. The cells were pelleted by centrifugation at 4,300 g for 40 min and pellets resuspended in 20 mM HEPES (pH 7.6), 200 mM NaCl and 0.1% Nonidet P-40, with a tablet of cOmplete, Mini EDTA-free protease inhibitor cocktail (Roche). After lysis by homogenization, the cell lysate was centrifuged at 10,000g for 30 min. The resulting clear lysate was passed through a 0.22 µm hydrophilic PVDF membrane filter (EMD Millipore) and subjected to a two-step purification using fast protein liquid chromatography (ÄKTA pure). First, the clear lysate was loaded onto a 5 mL HiTrap® SP HP column (Cytiva), and eluted with buffer containing 20 mM HEPES (pH 7.6), 10% glycerol, with a linear gradient from 200 mM NaCl to 1 M NaCl over 20 column volumes (CV). The fractions containing the tMeCP2 variant of interest were pooled and further purified with a 5 mL StrepTrap HP column (Cytiva). The column was washed with 5CV of wash buffer (20 mM HEPES (pH7.6), 500 mM NaCl, 10% glycerol) and eluted with wash buffer supplemented with 2.5 mM d-Desthiobiotin. The eluted desired fractions were combined, concentrated, and buffer exchanged into the final storage buffer (20 mM HEPES (pH7.6), 300 mM NaCl, 10% glycerol) using a PD-10 desalting column (Cytiva). For ZF5.3 tagged proteins, the storage buffer was supplemented with 100 µM $ZnCl_2$ and 1 mM dithiothreitol (DTT). After each column chromatography, the elution fractions were visualized by SDS-PAGE. The final purified recombinant *t*MeCP2 proteins were analyzed by mass spectrometry (Agilent 6530 QTOF LCMS) and western blot (anti-Strep-tag II antibody conjugated to horseradish peroxidase, IBA 2-1509-001) and stored at -80°C until needed. Protein concentrations were calculated based on the Beer-Lambert law $A = \varepsilon bc$, where A is the absorbance at 280 nm measured on a nanodrop spectrophotometer (ND-1000), ε is the extinction coefficient of the protein, and b is the optical path length (1 cm).

Sortase-mediated conjugation of fluorophore to proteins

The synthesis and purification of GGGK-Lissamine rhodamine B (Rho) were performed as previously described.[9] Before use in sortase-mediated labeling experiments, the peptide was resuspended in the minimum quantity of DMSO. The concentration of the solution was calculated based on the Beer-Lambert law $A = \varepsilon bc$, as described above. ε =112 000 M^{-1} cm^{-1}.[10]

Two variants of the hepta-mutant *Staphylococcus aureus* Sortase A (SrtA7m) were used to conjugate rhodamine to ZF5.3 tagged- and non-ZF5.3 tagged-*t*MeCP2 proteins due to different requirements in the downstream purification process after labeling. Two plasmids, one encoding

StrepTagII-SrtA7m-His6, the other His6-SUMO-SrtA7m, were transformed into *E. Coli* BL21(DE3) Gold cells (Agilent, #230132) and selected against appropriate bacterial resistance (kanamycin for StrepTagII-SrtA7m-His6 and carbenicillin for His6-SUMO-SrtA7m). Both proteins were purified as described previously[11] with slight modification. After expression, the cell pellet was resuspended in lysis buffer (20 mM HEPES (pH 7.6), 150 mM NaCl, 10% glycerol, and a tablet of cOmplete, Mini EDTA-free protease inhibitor cocktail (Roche)). After sonication, the clear lysate was incubated with 1.5 mL of TALON® metal affinity resin (Takara) pre-equilibrated with lysis buffer for 1 hr at 4 °C. The mixture was transferred to a 15 mL disposable column, and washed with 45 mL of wash buffer (20 mM HEPES (pH 7.6), 500 mM NaCl, 10% glycerol, 10 mM imidazole). The proteins were eluted in 15 mL of elution buffer (20 mM HEPES (pH 7.6), 150 mM NaCl, 10% glycerol, 200 mM imidazole), and analyzed on an SDS-PAGE gel. The desired fractions were dialyzed into 20 mM HEPES (pH7.6), 300 mM NaCl, 10 % glycerol overnight at 4 °C and stored at -80 °C until needed. Protein concentrations were determined by the Pierce™ 660 nm protein assay (ThermoFisher, 22660), using bovine serum albumin (BSA) as a standard.

For His6-SUMO-SrtA7m, the His6-SUMO tag was subsequently cleaved by incubating the protein with His6-SUMO protease purified as described previously[11] (2:1 molar ratio) at RT for 2 h. After incubating the reaction mixture with TALON® metal affinity resin for 1 hr at 4 °C, the cleaved SrtA7m protein was isolated from the His-tagged impurities in the flow-through and wash fractions. The desired fractions determined by SDS-PAGE gel were dialyzed into 20 mM HEPES (pH 7.6), 300 mM NaCl, 10% glycerol overnight at 4 °C, and stored at -80 °C until needed. Protein concentrations were determined by the Pierce™ 660 nm protein assay using BSA as a standard.

To generate fluorescently tagged proteins, 35 µM ZF5.3 tagged *t*MeCP2-LPETGG-Strep (ZF or T158M) were incubated with 75 µM SrtA7m and 200 µM GGGK-Lissamine rhodamine B (Rho) for 1hr at 37 °C, in the reaction buffer 20 mM HEPES (pH 7.6), 300 mM NaCl, 10% glycerol. To isolate ZF5.3-*t*MeCP2-Rho/ZF5.3-*t*MeCP2-T158M-Rho, the reaction mixtures were incubated with 1 mL of TALON® metal affinity resin pre-equilibrated with reaction buffer for 1 hr at 4 °C and transferred to a 15 mL disposable column. The column was washed with 30 mL of reaction buffer. The ZF5.3 fusion proteins binding to the TALON resin were eluted with 10 mL elution buffer (reaction buffer with 250 mM imidazole) and analyzed on an SDS-PAGE gel. The absence of unconjugated Rho in the purified protein was confirmed by fluorescence imaging. The desired fractions were combined and dialyzed into 20 mM HEPES (pH 7.6), 300 mM NaCl, 10% glycerol, 100 µM $ZnCl_2$ and 1 mM DTT overnight at 4 °C.

For fluorophore conjugation of non-ZF5.3-tagged tMeCP2, 35µM *t*MeCP2-LPETGG-Strep or Tat-*t*MeCP2-LPETGG-Strep were incubated with 75 µM StrepTagII-SrtA7m-His6 and 200 µM GGGK-Rho for 1 hr at 37 °C, in the reaction buffer 20 mM HEPES (pH 7.6), 300 mM NaCl, 10% glycerol. After incubation with 1 mL of TALON® metal affinity resin pre-equilibrated with the reaction buffer for 1 hr at 4°C, the reaction mixture was loaded to a 15 mL disposable

column. The proteins *t*MeCP2-Rho and Tat-*t*MeCP2-Rho were collected during the wash step with 20mL of reaction buffer. After visualization on an SDS-PAGE gel, the desired protein fractions were concentrated to 2.5 mL using a 3k MWCO centrifugal filter unit (Amicon®) and passed through a PD-10 desalting column (Cytiva) to remove unreacted free Rho in the solution.

The final purified proteins were analyzed by SDS-PAGE gel and mass spectrometry (Agilent Agilent 6530 QTOF LCMS) and stored at -80°C. The total protein concentrations were determined using the Pierce™ 660 nm protein assay and a standard curve generated by purified *t*MeCP2-LPETGG-Strep variants of known concentrations. The concentration of fluorophore-conjugated proteins was inferred from the Rho concentration, which was calculated based on the Beer-Lambert law $A = \varepsilon bc$, where A is the absorbance at 570 nm measured on a Nanodrop spectrophotometer, ε is the extinction coefficient of Rho in water (112000 M^{-1} cm^{-1}).[10] The efficiency of Rho conjugation to *t*MeCP2 was calculated by dividing the Rho concentration by the total protein concentration.

Supplementary Methods 2. NIH3T3 nuclear lysate isolation

To obtain the nuclear lysate, the intact nuclei were first prepared based on a protocol outlined by Millipore Sigma (https://www.sigmaaldrich.com/US/en/technical-documents/protocol/protein-biology/protein-lysis-and-extraction/extraction-from-tissue) with slight modifications. Four 150-mm dishes of NIH3T3 cells were grown to 80% confluency (~5.5 million per plate). After washing twice with DPBS, the cells were lifted using 8 mL of enzyme-free cell dissociation buffer (Gibco) for 5 min at 37 °C, 5% CO_2. The cells were washed with 10 mL DPBS and transferred into a centrifuge tube to spin at 200 g, 3 min. The cell pellets were pooled together in a microcentrifuge tube and the packed cell volume (PCV) was estimated (~150-200 µL). After resuspending the pellet in 300 µL hypotonic lysis buffer (10 mM HEPES, pH 7.9, 10 mM KCl, 1.5 mM $MgCl_2$, 1 mM DTT, protease inhibitor (Roche)), the mixture was spun at 100 g for 5 min for initial cell swelling. The supernatant was discarded and the pellet was resuspended in 300 µL hypotonic lysis buffer and placed on ice for 10 min for further swelling. The mixture was transferred to a 2 mL Dounce homogenizer and ground on ice slowly with 45 up-and-down strokes using a type B pestle. Lysis of the cell membrane was checked to be 80-90% complete under the microscope using Trypan Blue as the indicator. The lysate was centrifuged for 5 min at 5,000 x g and the supernatant (cytoplasmic fraction) was separated from the crude nuclei pellet (~100 µL PCV).

The soluble nuclear proteins were isolated following the methods reported by Lyst *et al*[7] with slight modifications. The crude nuclei pellet was first dissolved in 100 µL of low salt extraction buffer (20 mM HEPES, pH 7.6, 10 mM KCl, 1 mM $MgCl_2$, 0.1% Triton X-100 (vol/vol), protease inhibitors (Roche), 15 mM BME) and dounced with 20 up-and-down strokes using a type B pestle. The lysate was then transferred to a microcentrifuge tube along with an extra 100 µL low salt extraction buffer added to wash the dounce vessel. 250 units of benzonase (Sigma)

per 10^7 nuclei was added to the dounced nuclei for 10 min at RT. High salt extraction buffer (low salt + 1 M NaCl) was added dropwise to achieve a final NaCl concentration of 350 mM and the mixture was incubated on a rotating wheel for 45 min at 4℃. The final soluble nuclear supernatant was obtained by centrifuging at 18,000 g for 15 min.

To preserve the interaction of *t*MeCP2 with the NCoR/SMRT complex,[7] the nuclear supernatant was buffer exchanged for four times with the coIP buffer (20 mM HEPES, pH 7.6, 10 mM KCl, 150 mM NaCl, 1 mM $MgCl_2$, 0.1% Triton X-100 (vol/vol), protease inhibitors (Roche), 15 mM BME) using a 3k MWCO concentrator (Amicon). The final concentration of the nuclear lysate was determined by the Pierce™ 660 nm protein assay, using BSA as standards and diluted to ~1 mg/mL with the coIP buffer.

Supplementary Methods 3. LC/MS/MS Analysis

Mass spectrometry was performed by the Proteomics/Mass Spectrometry Laboratory at UC Berkeley. A nano LC column was packed in a 100 μm inner diameter glass capillary with an emitter tip. The column consisted of 10 cm of Polaris c18 5 μm packing material (Varian). The column was loaded by use of a pressure bomb and washed extensively with buffer A (5% acetonitrile/ 0.02% heptaflurobutyric acid (HBFA)). The column was then directly coupled to an electrospray ionization source mounted on a Thermo-Fisher LTQ XL linear ion trap mass spectrometer. An Agilent 1200 HPLC equipped with a split line so as to deliver a flow rate of 300 nl/min was used for chromatography. Peptides were eluted using a 90 min. gradient from buffer A to 60% Buffer B (80% acetonitrile/ 0.02% HBFA).

Protein identification and quantification were done with Integrated Proteomics Pipeline (IP2, Integrated Proteomics Applications, Inc. San Diego, CA) using ProLuCID/Sequest, DTASelect2 and Census.[14–17] Tandem mass spectra were extracted into ms1 and ms2 files from raw files using RawExtractor[18] and were searched against the *E. Coli* protein database plus sequences of common contaminants with the engineered sequence of the experimental protein added. This database was concatenated to a decoy database in which the sequence for each entry in the original database was reversed.[19] LTQ data was searched with 3000.0 milli-amu precursor tolerance and the fragment ions were restricted to a 600.0 ppm tolerance. All searches were parallelized and searched on the VJC proteomics cluster. Search space included all fully tryptic peptide candidates with no missed cleavage restrictions. Carbamidomethylation (+57.02146) of cysteine was considered a static modification. We required 1 peptide per protein and both tryptic termini for each peptide identification. The ProLuCID search results were assembled and filtered using the DTASelect program[15,16] with a peptide false discovery rate (FDR) of 0.001 for single peptides and a peptide FDR of 0.005 for additional peptides for the same protein. Under such filtering conditions, the estimated false discovery rate was zero for the datasets used.

Supplementary Methods 4. Isolation of Primary Cortical Neurons

On dissection day, 50 mL of Complete Neurobasal Media (CNB) were freshly prepared starting from 48.5 mL Neurobasal Media (Invitrogen, 21103049), 1 mL B27 supplement (Invitrogen, 17504-044), 250 μL GlutaMax (Invitrogen, 35050-061) and 250 μL Pen/Strep antibiotics (Invitrogen, 15140-122). The newly prepared CNB was equilibrated in a regular cell culture incubator for 1 hour before use. Neurons were dissociated from cortical tissue with the Papain Dissociation System Kit, prepared according to manufacturer instructions (Worthington, LK003153). Both the papain (~ 20 units/mL papain and 0.005% DNase) and the inhibitor solutions were equilibrated in a regular cell culture incubator for 15 minutes and filtered (0.22 μm) prior to use. Cortices were dissected from brains under a stereo microscope (AmScope, SM-1TSZZ-144S-10M) with sterile surgical tools in ice-cold Hanks' Balanced Salt Solution (HBSS) with no Ca^{2+} nor Mg^{2+} (Invitrogen, 14110-172) added of Pen/Strep. Meninges were gently pulled away from cortices and the dissected tissue was immediately transferred to 500 mL of equilibrated CNB in a 1.5-mL microcentrifuge tube, where it was coarsely cut with tweezers and left to settle at the bottom of the tube. CNB was carefully removed from the settled tissue and replaced with 500 μL of pre-warmed papain solution. Cortices were digested for 45 minutes in a thermomixer set to 37 °C and 1,000 rpm. We left the dissociated tissue settle at the bottom of the tube and replaced the papain solution with 500 μL of pre-warmed inhibitor, incubating for 5 minutes in a thermomixer set to 37 °C and 1,000 rpm. The inhibitor was then replaced with 1 mL of equilibrated CNB. The dissociated tissue was triturated to a single-cell suspension pipetting vigorously ~25 times up and down with a P1000 pipette. Cells were passed through a 40-mm cell strainer (pre-washed twice with 1 mL of CNB media) to remove cell clumps. After passing the cells, the filter was washed again with 1 mL CNB. Cells were mixed well and counted in Trypan Blue (Sigma, T8154-20ML) with a hemocytometer. Cells were plated in 12-well plates onto 18-mm high precision microscope cover glasses No. 1.5H (Marienfeld, distributed by Azer, ES0117580), which were plasma-cleaned (Diener electronic FEMTO plasma system) prior to coating for 1 hour at 37 °C with a poly-D-lysine solution (2.5 mg/mL final in water; Sigma-Aldrich P6407). After coating, cover glasses were rinsed 3 times with sterile water and left in the laminar hood to dry completely before seeding cells onto them (seeding density: $6x10^5$ cells per well). Neurons were allowed to attach to the cover glasses for 2 hours in a regular cell culture incubator before the media was gently replaced with fresh one to remove unattached glial cells. Dissected neurons were matured in culture for 13 days prior to staining and imaging, changing half of their media every 4 days.

Supplementary Note 1. FCS Data Analysis

The raw autocorrelation and countrate data obtained from FCS measurements were analyzed using a custom MATLAB script as previously described[8,9] with some slight modification for measurements in the nucleus. From the ten curves collected at each point, low-quality curves due to diffusional artifacts were discarded. The remaining curves were averaged and fitted to a 3D diffusion equation (eq 1, *in vitro* measurements) or a 3D anomalous diffusion equation (eq 2, *in*

cellulo cytosolic measurements) and a two-component 3D diffusion equation (eq 3, *in cellulo* nuclear measurements). To select for the equation that more accurately fits the *in cellulo* nuclear measurements, three fitting equations (eq 2, eq 3 and eq 4) were used to fit the data collected for different *t*MeCP2-Rho variants at different treatment concentrations (20-30 points for each variant). Chi-square values were calculated and compared for each fitting to confirm that eq 3 is the most appropriate model for fitting the nuclear FCS measurements (**Table S4**). Adding an anomaly parameter for the fast fraction (eq 5) made the values of the fit parameters more uncertain and hard to interpret. While other models like the reaction-dominant model have been applied to chromatin-binding proteins, a two-component diffusion model can provide reasonably accurate fitting for measurements with high free fractions[20] (Free fraction > 65% for *t*MeCP2-Rho in both Saos-2 and CHO-K1 cells, **Table S2, S3**). We have corrected a typo in eq 2 present in the previous MATLAB script and the updated script can be accessed at https://github.com/schepartzlab/FCS.

$$G(\tau) = \left(\frac{1}{N}\right)\frac{1}{\left(1+\frac{\tau}{\tau_{diff}}\right)\sqrt{\left(1+s^2\frac{\tau}{\tau_{diff}}\right)}} \quad (1)$$

$$G(\tau) = \left(\frac{1}{N}\right)\frac{1}{\left(1+\left(\frac{\tau}{\tau_{diff}}\right)^{\alpha}\right)\sqrt{1+s^2\left(\frac{\tau}{\tau_{diff}}\right)^{\alpha}}} + G(\infty) \quad (2)$$

$$G(\tau) = \frac{1}{N}\left[F_{fast}\frac{1}{\left(1+\frac{\tau}{\tau_{diff}^{fast}}\right)\sqrt{\left(1+s^2\frac{\tau}{\tau_{diff}^{fast}}\right)}}\right]\left[(1-F_{fast})\frac{1}{\left(1+\left(\frac{\tau}{\tau_{diff}^{slow}}\right)^{\alpha}\right)\sqrt{1+s^2\left(\frac{\tau}{\tau_{diff}}\right)^{\alpha}}}\right] \quad (3)$$

$$G(\tau) = \frac{1}{N}\left[F_{fast}\frac{1}{\left(1+\frac{\tau}{\tau_{diff}^{fast}}\right)\sqrt{\left(1+s^2\frac{\tau}{\tau_{diff}^{fast}}\right)}}\right]\left[(1-F_{fast})\frac{1}{\left(1+\frac{\tau}{\tau_{diff}^{slow}}\right)\sqrt{\left(1+s^2\frac{\tau}{\tau_{diff}^{slow}}\right)}}\right] \quad (4)$$

$$G(\tau) = \frac{1}{N}\left[F_{fast}\frac{1}{\left(1+\left(\frac{\tau}{\tau_{diff}^{fast}}\right)^{\alpha_{fast}}\right)\sqrt{1+s^2\left(\frac{\tau}{\tau_{diff}^{fast}}\right)^{\alpha_{fast}}}}\right]\left[(1-F_{fast})\frac{1}{\left(1+\left(\frac{\tau}{\tau_{diff}^{slow}}\right)^{\alpha_{slow}}\right)\sqrt{1+s^2\left(\frac{\tau}{\tau_{diff}^{slow}}\right)^{\alpha_{slow}}}}\right]$$

(5)

From the fitting, a set of parameters specific to individual measurements were obtained, including the diffusion time of molecules (τ_{diff}), the average number of diffusing molecules in the focal volume (N), anomalous diffusion coefficients (α) and the fraction that are fast diffusing (F_{fast}). The resulting data were filtered as described previously.[21] For *in cellulo* nuclear measurements, we further select by retaining data that have a reasonable fitting chi-square between 1-15. Outliers were removed using the ROUT method (Q = 0.1%). For the analysis of fitting parameters from *in cellulo* nuclear measurements in **Table S2, S3**, we excluded the fittings that fail to identify a second component by generating a τ_{diff}^{slow} equal to the preset lower limit of 1.5 ms.

Determination of the effective confocal volume (V_{eff}) from in vitro measurements

The autocorrelation curves obtained from the Alexa 594 standards were fitted to a 3D diffusion equation (eq 1):

$$G(\tau) = (\frac{1}{N})\frac{1}{(1+\frac{\tau}{\tau_{diff}})\sqrt{(1+s^2\frac{\tau}{\tau_{diff}})}} \quad (1)$$

where N= the average number of diffusing molecules in the focal volume (V_{eff}), τ_{diff}= the average diffusion time that a molecule takes to transit the volume, *s* = structural factor, which is the ratio of the radial to axial dimensions of the focal volume. *s* was determined by fitting the autocorrelation function of Alexa 594 in water at 25°C to be 0.17, and these values were used for all following analysis.

To calculate the V_{eff}, we first calculated the radius of the confocal volume ω_1 using equation 6:

$$\omega_1 = \sqrt{4 \cdot D \cdot \tau_{diff}} \quad (6)$$

where τ_{diff} was obtained from the fitting above and D = the diffusion coefficient of Alexa 594 at 37°C, previously determined to be $5.20 \times 10^{-6} cm^2 s^{-1}$.[9]

Then V_{eff} can be calculated based on equation 7:

$$V_{eff} = \pi^{\frac{3}{2}} \cdot w_1^3 \cdot \frac{1}{s} \quad (7)$$

The resulting V_{eff} of each experiment ranged from 0.3-0.4 femtoliters.

Calculation of cytosolic and nuclear concentrations from in cellulo measurements

The autocorrelation curves obtained from the *in cellulo* cytosolic measurements were fitted to a 3D anomalous diffusion equation (equation 2):

$$G(\tau) = (\frac{1}{N})\frac{1}{(1+\frac{\tau}{\tau_{diff}})^{\alpha}\sqrt{1+s^2(\frac{\tau}{\tau_{diff}})^{\alpha}}} + G(\infty) \quad (2)$$

The autocorrelation curves obtained from the *in cellulo* nuclear measurements were fitted to a two-component 3D free+anomalous diffusion equation (equation 3):

$$G(\tau) = \frac{1}{N}[F_{fast}\frac{1}{(1+\frac{\tau}{\tau_{diff}^{fast}})\sqrt{(1+s^2\frac{\tau}{\tau_{diff}^{fast}})}}][(1-F_{fast})\frac{1}{(1+\frac{\tau}{\tau_{diff}^{slow}})^{\alpha}\sqrt{1+s^2(\frac{\tau}{\tau_{diff}})^{\alpha}}}] \quad (3)$$

The cellular concentrations (C) can be calculated based on equation 8:

$$C = \frac{N}{N_A \cdot V_{eff}} \quad (8)$$

where N_A = Avogadro's number, $6.023 \times 10^{23} mol^{-1}$.

The concentrations obtained above represent the concentrations of the fluorescently tagged proteins in cells. Because the sortase-mediated labeling reaction did not generate a homogenous solution of Rho conjugated *t*MeCP2, we adjusted the cellular concentrations with a correction factor to account for the proteins that entered the cells but cannot be detected without a fluorophore (equation 9).

$$C_{corr} = C \frac{[tMeCP2]}{[tMeCP2-Rho]} \quad (9)$$

where C_{corr} = the corrected cellular concentration, [*t*MeCP2] = total protein concentration (Rho-conjugated and unconjugated) in a sample delivered to cells, [*t*MeCP2-Rho] = the concentration of Rho-conjugated tMeCP2 in a sample delivered to cells.

References

(1) Greenfield, N. J. Using Circular Dichroism Spectra to Estimate Protein Secondary Structure. *Nat. Protoc.* **2006**, *1* (6), 2876–2890. https://doi.org/10.1038/nprot.2006.202.

(2) Ghosh, R. P.; Nikitina, T.; Horowitz-Scherer, R. A.; Gierasch, L. M.; Uversky, V. N.; Hite, K.; Hansen, J. C.; Woodcock, C. L. Unique Physical Properties and Interactions of the Domains of Methylated DNA Binding Protein 2. *Biochemistry* **2010**, *49* (20), 4395–4410. https://doi.org/10.1021/bi9019753.

(3) Ghosh, R. P.; Horowitz-Scherer, R. A.; Nikitina, T.; Gierasch, L. M.; Woodcock, C. L. Rett Syndrome-Causing Mutations in Human MeCP2 Result in Diverse Structural Changes That Impact Folding and DNA Interactions. *J. Biol. Chem.* **2008**, *283* (29), 20523. https://doi.org/10.1074/jbc.M803021200.

(4) Khrapunov, S.; Warren, C.; Cheng, H.; Berko, E. R.; Greally, J. M.; Brenowitz, M. Unusual Characteristics of the DNA Binding Domain of Epigenetic Regulatory Protein MeCP2 Determine Its Binding Specificity. *Biochemistry* **2014**, *53* (21), 3379–3391. https://doi.org/10.1021/bi500424z.

(5) Ghosh, R. P.; Horowitz-Scherer, R. A.; Nikitina, T.; Shlyakhtenko, L. S.; Woodcock, C. L. MeCP2 Binds Cooperatively to Its Substrate and Competes with Histone H1 for Chromatin Binding Sites. *Mol. Cell. Biol.* **2010**, *30* (19), 4656–4670. https://doi.org/10.1128/MCB.00379-10.

(6) Khrapunov, S.; Tao, Y.; Cheng, H.; Padlan, C.; Harris, R.; Galanopoulou, A. S.; Greally, J. M.; Girvin, M. E.; Brenowitz, M. MeCP2 Binding Cooperativity Inhibits DNA Modification-Specific Recognition. *Biochemistry* **2016**, *55* (31), 4275–4285. https://doi.org/10.1021/acs.biochem.6b00451.

(7) Lyst, M. J.; Ekiert, R.; Ebert, D. H.; Merusi, C.; Nowak, J.; Selfridge, J.; Guy, J.; Kastan, N. R.; Robinson, N. D.; de Lima Alves, F.; et al. Rett Syndrome Mutations Abolish the Interaction of MeCP2 with the NCoR/SMRT Co-Repressor. *Nat. Neurosci.* **2013**, *16* (7), 898–902. https://doi.org/10.1038/nn.3434.

(8) Knox, S. L.; Steinauer, A.; Alpha-Cobb, G.; Trexler, A.; Rhoades, E.; Schepartz, A. Quantification of Protein Delivery in Live Cells Using Fluorescence Correlation Spectroscopy. *Methods Enzymol.* **2020**, *641*, 477–505. https://doi.org/10.1016/bs.mie.2020.05.007.

(9) Steinauer, A.; LaRochelle, J. R.; Knox, S. L.; Wissner, R. F.; Berry, S.; Schepartz, A. HOPS-Dependent Endosomal Fusion Required for Efficient Cytosolic Delivery of Therapeutic Peptides and Small Proteins. *Proc. Natl. Acad. Sci.* **2019**, *116* (2), 512–521. https://doi.org/10.1073/pnas.1812044116.

(10) Wissner, R. F.; Steinauer, A.; Knox, S. L.; Thompson, A. D.; Schepartz, A. Fluorescence Correlation Spectroscopy Reveals Efficient Cytosolic Delivery of Protein Cargo by Cell-Permeant Miniature Proteins. *ACS Cent. Sci.* **2018**, *4* (10), 1379–1393. https://doi.org/10.1021/acscentsci.8b00446.

(11) Knox, S. L.; Wissner, R.; Piszkiewicz, S.; Schepartz, A. Cytosolic Delivery of

Argininosuccinate Synthetase Using a Cell-Permeant Miniature Protein. *ACS Cent. Sci.* **2021**, *7* (4), 641–649. https://doi.org/10.1021/acscentsci.0c01603.

(12) Eccles, L. J.; Lomax, M. E.; O'Neill, P. Hierarchy of Lesion Processing Governs the Repair, Double-Strand Break Formation and Mutability of Three-Lesion Clustered DNA Damage. *Nucleic Acids Res.* **2010**, *38* (4), 1123–1134. https://doi.org/10.1093/nar/gkp1070.

(13) Zhao, Z. W.; White, M. D.; Alvarez, Y. D.; Zenker, J.; Bissiere, S.; Plachta, N. Quantifying Transcription Factor–DNA Binding in Single Cells in Vivo with Photoactivatable Fluorescence Correlation Spectroscopy. *Nat. Protoc.* **2017**, *12* (7), 1458–1471. https://doi.org/10.1038/nprot.2017.051.

(14) Xu, T.; Park, S. K.; Venable, J. D.; Wohlschlegel, J. A.; Diedrich, J. K.; Cociorva, D.; Lu, B.; Liao, L.; Hewel, J.; Han, X.; et al. ProLuCID: An Improved SEQUEST-like Algorithm with Enhanced Sensitivity and Specificity. *J. Proteomics* **2015**, *129*, 16–24. https://doi.org/10.1016/j.jprot.2015.07.001.

(15) Cociorva, D.; L Tabb, D.; Yates, J. R. Validation of Tandem Mass Spectrometry Database Search Results Using DTASelect. *Curr. Protoc. Bioinforma.* **2007**, *Chapter 13*, Unit 13.4. https://doi.org/10.1002/0471250953.bi1304s16.

(16) Tabb, D. L.; McDonald, W. H.; Yates, J. R. DTASelect and Contrast: Tools for Assembling and Comparing Protein Identifications from Shotgun Proteomics. *J. Proteome Res.* **2002**, *1* (1), 21–26. https://doi.org/10.1021/pr015504q.

(17) Park, S. K.; Venable, J. D.; Xu, T.; Yates, J. R. A Quantitative Analysis Software Tool for Mass Spectrometry–Based Proteomics. *Nat. Methods* **2008**, *5* (4), 319–322. https://doi.org/10.1038/nmeth.1195.

(18) McDonald, W. H.; Tabb, D. L.; Sadygov, R. G.; MacCoss, M. J.; Venable, J.; Graumann, J.; Johnson, J. R.; Cociorva, D.; Yates, J. R. MS1, MS2, and SQT-Three Unified, Compact, and Easily Parsed File Formats for the Storage of Shotgun Proteomic Spectra and Identifications. *Rapid Commun. Mass Spectrom. RCM* **2004**, *18* (18), 2162–2168. https://doi.org/10.1002/rcm.1603.

(19) Peng, J.; Elias, J. E.; Thoreen, C. C.; Licklider, L. J.; Gygi, S. P. Evaluation of Multidimensional Chromatography Coupled with Tandem Mass Spectrometry (LC/LC−MS/MS) for Large-Scale Protein Analysis: The Yeast Proteome. *J. Proteome Res.* **2003**, *2* (1), 43–50. https://doi.org/10.1021/pr025556v.

(20) Michelman-Ribeiro, A.; Mazza, D.; Rosales, T.; Stasevich, T. J.; Boukari, H.; Rishi, V.; Vinson, C.; Knutson, J. R.; McNally, J. G. Direct Measurement of Association and Dissociation Rates of DNA Binding in Live Cells by Fluorescence Correlation Spectroscopy. *Biophys. J.* **2009**, *97* (1), 337–346. https://doi.org/10.1016/j.bpj.2009.04.027.

(21) LaRochelle, J. R.; Cobb, G. B.; Steinauer, A.; Rhoades, E.; Schepartz, A. Fluorescence Correlation Spectroscopy Reveals Highly Efficient Cytosolic Delivery of Certain Penta-Arg Proteins and Stapled Peptides. *J. Am. Chem. Soc.* **2015**, *137* (7), 2536–2541. https://doi.org/10.1021/ja510391n.
